# Intraguild Interactions Drive the Dynamics of a Complex Community of Globally Invasive Ant Species

**DOI:** 10.1111/mec.70464

**Published:** 2026-07-09

**Authors:** Maximillian P. T. G. Tercel, William O. C. Symondson, Jordan P. Cuff, Nik C. Cole, Martine Goder, Kevin Ruhomaun, Vikash Tatayah, Ian P. Vaughan

**Affiliations:** ^1^ School of Biosciences Cardiff University Cardiff UK; ^2^ Durrell Wildlife Conservation Trust, Les Augrès Manor Trinity Jersey; ^3^ School of Natural and Environmental Sciences Newcastle University Newcastle upon Tyne UK; ^4^ Mauritian Wildlife Foundation Vacoas Mauritius; ^5^ National Parks & Conservation Service Ministry of Agro Industry & Food Security Head Office Mauritius

**Keywords:** dietary metabarcoding, Formicidae, hypergraphs, interference and exploitation competition, non‐native ants, trophic interactions

## Abstract

Invasive species adversely affect biodiversity, but little is known about how ecologically similar invaders interact. Such intraguild interactions may drive non‐native community dynamics and their broader impacts on biodiversity. In native systems, intraguild interactions are widespread strong eco‐evolutionary pressures; understanding whether similar patterns occur among invaders is key to predicting invasion outcomes. We investigated the role of intraguild predation in a community of non‐native ant species inhabiting a tropical island with no native ants. Using dietary metabarcoding, we quantified intraguild predation, applied null models to test dietary and spatial preferences, conducted food bait experiments to assess competition for resources, and quantified potential competition strength using a hypergraph network approach. Metabarcoding 755 ants across 12 species revealed that ~50% of prey detections represented ant‐ant consumption. Ants preferentially consumed other ants over native arthropods but only once the globally invasive big‐headed ant, *Pheidole megacephala* , was removed from the null model analysis due to its hyperabundance. Additionally, 35% of food resources were identified as foci for potential interspecific exploitation where ant species spatially co‐occurred. *Pheidole megacephala* occupied a key structural role in the community: it frequently competed with others for prey, was consumed less often than expected given its abundance, and was spatially avoided by other ants. Overall, interference competition was dominated by *P. megacephala* , but exploitation competition was high across the community. These findings suggest that intraguild interactions strongly structure invaded communities, influencing the ecological impact of multiple invaders within the same guild.

## 1 Introduction

Invasive species can be a major threat to biodiversity (Clavero and Garcia‐Berthou 2005; Bradley et al. 2019) and are expanding due to the globalisation of trade and travel (Hulme 2009). While their adverse effects on native species and ecosystems are well documented, interactions among co‐occurring non‐native species remain comparatively underexplored. Interactions between ecologically similar (hereafter ‘intraguild’) non‐native species are likely to be especially important because competitive and predatory processes are often tightly linked within ecological guilds (Polis et al. 1989). Intraguild predation occurs when species compete for shared resources while also consuming one another—a dynamic distinct from competition or predation alone. Intraguild predators therefore benefit nutritionally from consuming their intraguild prey whilst also eliminating a potential competitor (Polis et al. 1989). These interactions can govern community structure, resource partitioning, species coexistence, and the ecological impact of invasive species.

In native systems, intraguild interactions are widespread and represent major eco‐evolutionary pressures (Polis et al. 1989; Arim and Marquet 2004), but it remains unclear to what degree these interactions shape invaded ecosystems where species may lack co‐evolved traits. In native communities, intraguild interactions influence the distribution, abundance and evolution of species within the guild (Arim and Marquet 2004), and the coexistence of intraguild predators and prey is thought to depend on asymmetries in predation ability and resource acquisition (Mylius et al. 2001; Arim and Marquet 2004; Björklund et al. 2016). Intraguild predators will often clear the area immediately around their territory or nest to consume potential competitors, and this behaviour is seen in many taxa, such as corals (Thomason and Brown 1986), and canids, mustelids, and bears (Mabelis 1983). Similarly, intraguild predators might exclude intraguild prey species from resources, but prey can coexist with their predators if they can extract other resources from the environment more effectively. Intraguild predation can also be mutual, where species *A* and *B* actively prey upon one another in different scenarios (Polis et al. 1989); for example, if species *A* and *B* are social animals, *A* only preys on *B* when it outnumbers it, but the relationship is reversed if they meet singly or *B* outnumbers *A*. For taxa where intraguild predation has been a significant selective pressure, intraguild prey may develop behavioural strategies or physiological adaptations that reduce the likelihood of being predated or even facilitate opportunistic predation of their predators (Folt and Goldman 1981).

Intraguild predation is usually studied using a simple three species system: an intraguild predator, intraguild prey, and a resource species (Polis et al. 1989; Björklund et al. 2016). Communities structured by intraguild interactions are predicted to exhibit a high incidence of intraguild predation by each predator (Polis et al. 1989; Traugott et al. 2012), leading to a complex set of trophic interactions. This can be tested by analysing the incidence of intraguild predation as a proportion of the total trophic interactions for each species of interest. In native communities, intraguild predators may also disproportionately select prey within, rather than outside, the guild (Polis et al. 1989; Arim and Marquet 2004), but this may not be the case for non‐native species. There are also likely to be non‐random local distribution patterns, where intraguild prey are found less frequently in areas with their predator, while the behavioural traits of some species may determine how other intraguild species can access non‐intraguild food resources. Here, we frame intraguild interactions in non‐native communities within a conceptual model consisting of three core mechanisms: (1) trophic interactions in the form of intraguild consumption, (2) interference and exploitation competition over shared resources and (3) spatial or behavioural exclusion driven by ecological dominance. We hypothesise that such interactions are not only common but also central to the assembly and composition of multi‐invader communities. This framing allows us to test whether dominant non‐native species act as agents of community structuring, constraining the abundance, behaviour, and diet of other invaders within the same guild.

Non‐native ants are ideal for investigating these concepts. They are globally widespread (Holway et al. 2002), can reduce native biodiversity substantially (Tercel et al. 2023) and multiple species of non‐native ants are typically found in invaded areas. In native communities, interactions between ants are considered foundational to community structure (Savolainen and Vepsäläinen 1988). If similar dynamics occur among non‐native ants, it suggests that intraguild interactions are a more ‘universal’ feature of ant ecology, regardless of biogeographic origin.

Here we examine the importance of intraguild predation within a community of 18 non‐native ant species on an islet in the Indian Ocean (Tercel et al. 2025). The ant community consists of non‐native species, which are either widely thought to be invasive or otherwise pantropically distributed. Using a combination of dietary DNA metabarcoding, conventional myrmecological sampling, and food bait experiments, we use this community to test the following three hypotheses:

1. Intraguild predation is prevalent across the ant community, both as the proportion of ant species engaging in it and the frequency of intraguild prey in their diets.
2. Intraguild trophic interactions and spatial cooccurrence patterns are non‐random, shaped by dominance hierarchies, life history traits, and species‐specific prey preferences, consistent with partitioning driven by competition and/or predation.
3. Ecologically dominant species modulate access to food resources and space, are consumed less frequently as intraguild prey and engage more in competitive interactions, thus regulating community composition.

## 2 Methods

### 2.1 Study Site and Ant Community

**FIGURE 1:**
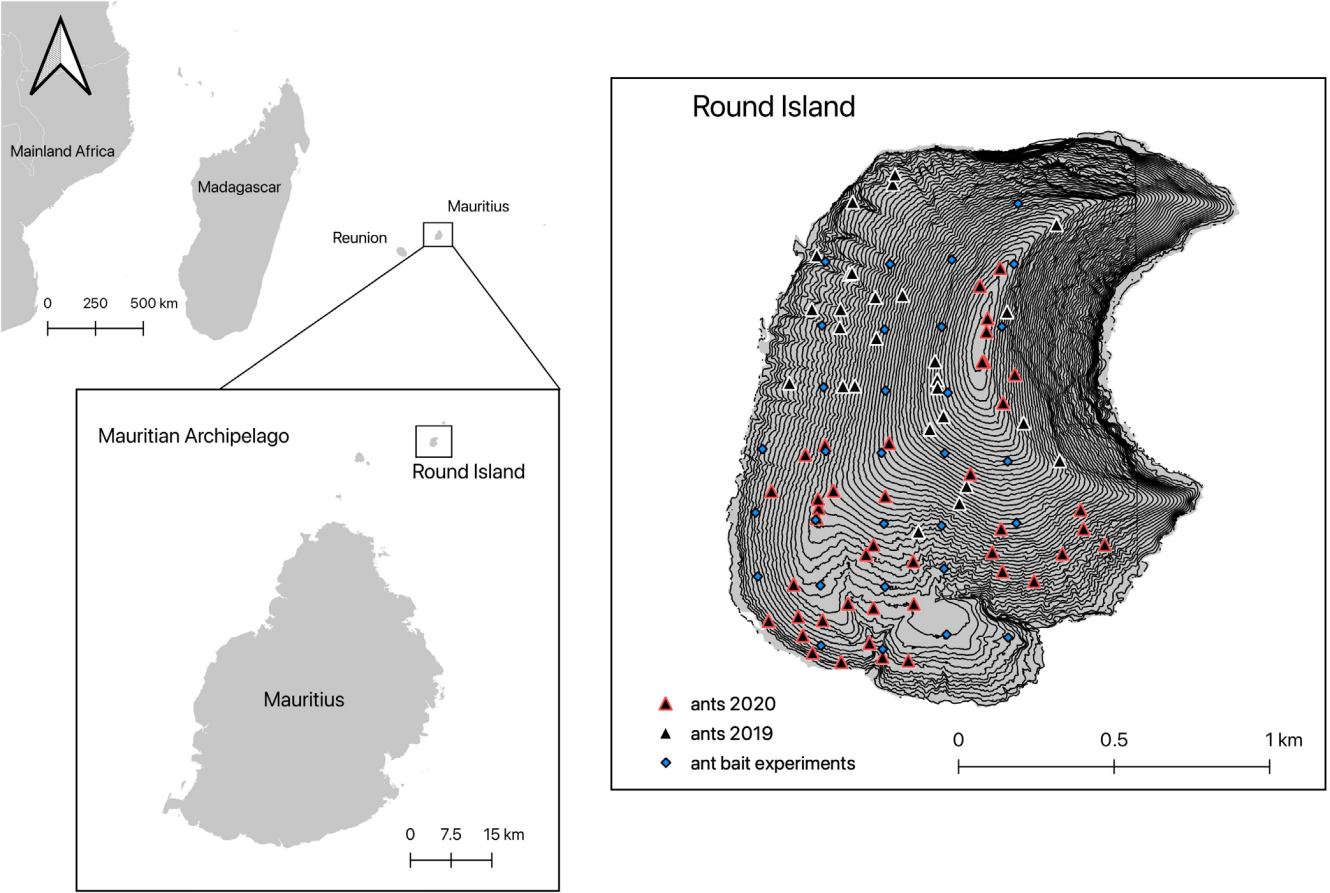
The position of Round Island in the Indian Ocean. The map on the right shows the sampling locations for ants on Round Island. Triangles denote randomly generated 4 m^2^ quadrats used to sample ants: white borders = sampled only in 2019, red borders = sampled in 2019 and 2020. Blue diamonds indicate where ant baiting trials were conducted. The topography of Round Island is shown by 5 m contour lines.

Round Island (Figure 1) is a 219 ha basaltic cone that reaches 280 m above sea level and represents the last remnant of native lowland palm forest within the Mascarenes (Cheke and Hume 2008). It is located 22.5 km north‐east of Mauritius in the Indian Ocean. The island suffered severe habitat destruction because of introduced goats and rabbits, which were eradicated in 1979 and 1986 respectively, and native habitat has been recovering since (Cheke and Hume 2008). Round Island has never suffered from invasion by non‐native predatory mammals, such as rats and therefore hosts many endemic species extirpated from other islands and mainland Mauritius (Cheke and Hume 2008; Tercel et al. 2024).

Broad dry and wet seasons exist in Mauritius (Senapathi et al. 2009). The dry season begins in May, with low rainfall, a mean air temperature of ~20.5°C and stronger winds. The driest months are September and October. The wet season begins in December, with much more frequent rainfall, a mean air temperature of ~24.5°C and minimal wind. The wettest months are January and February (Senapathi et al. 2009).

The ant fauna comprises 18 species, 17 of which are pantropically distributed non‐native ants found on both mainland and island systems (Holway et al. 2002), while the origin of the remaining species (*Hypoponera* mu03) is uncertain. Overall, the invasion history of ants on Round Island is poorly understood. Some of the records detailed here are new species records for Round Island arising from this study. See Appendix S1 (Table S1) for more information on the history of ant invasions on Round Island.

All individual ants collected during the study were identified to genus using Fisher and Bolton (2016), and to species level using Bolton (2007), Seifert (2002), Heterick (2006), LaPolla et al. (2011), and Sarnat et al. (2015). Identifications were further confirmed by checking the taxon assigned to the most abundant read count (which is most often associated with the focal predator; Cuff et al. 2023) in the COI sequencing results from the universal animal primers we used. These reads were later removed ahead of statistical analyses.

### 2.2 Sample Collection

We randomly generated 4 m^2^ quadrats over Round Island to sample the invertebrate community and collect ants for dietary metabarcoding. To sample epigeal invertebrates, a 150 mL pitfall trap was placed in the centre of each quadrat and half‐filled with 50% ethanol. Traps were collected after approximately 48 h and transferred into 15 mL universal collection tubes. Some of the ground surface of Round Island is bare rock, where traditional pitfall trapping is impossible. For quadrats that were generated in these areas, we designed pitfall traps with 12 cm canvas skirting radiating from the rim that were fixed to the substrate using masonry nails (Appendix S1; Figure S1). Seventy‐seven pitfall samples were collected, 42 in 2019 and 35 in 2020, and stored at room temperature until they could be refrigerated at −20°C. Table S2 contains replication information for each ecological factor we aimed to examine in this study (Appendix S1, Table S2).

Ants for dietary analysis were collected between September 2019 and March 2020 from each 4 m^2^ quadrat after invertebrate community samples were collected. The area was scoured for ant nests by hand searching, digging into soil, checking vegetation, and disturbing and sifting through leaf litter. Once a nest was found, pooters were used to collect and transfer ants into 15 mL collection tubes. Ants were identified to species or morphospecies in the field and each ant species was collected separately. Each collection tube contained a single ant species from a single colony and the quadrat was scoured until no additional species could be found. Ants were killed by freezing and were preserved in 100% ethanol. Samples were stored at −5°C on Round Island before being moved to −20°C storage on mainland Mauritius and subsequently to −80°C at Cardiff University, UK.

### 2.3 Food Bait Trials

Baiting trials were conducted over 28 days in August 2019 during the hours of 08:00–11:30 and 14:00–17:30 to observe the foraging behaviour of the Round Island ant community. Baits were a 15 mL mixture of honey, mashed mackerel, and peanut butter in a 1:1:1 ratio. They were placed on 25 cm^2^ squares of kitchen roll, ensuring the bait was accessible to ants from all directions and monitored for 30 min. We placed the bait on a relatively level area and recorded the time it took for each ant species to find the bait, evidenced by interaction with the bait through antennation or mastication. We measured the time taken for ant species to recruit 10 simultaneously foraging workers to the bait and any expulsion events where one species was removed from a bait by another. Baiting locations were situated on a 200 × 200 m grid over the accessible areas of Round Island, and a bait experiment was conducted at points where lines intersected (*n* = 85). Ants were identified in the field to morphospecies, and then at least one individual from each morphospecies attending was collected at the end of the experiment to ensure accurate species identification. We restricted analyses to the three most abundant ant species over the island, *Brachymyrmex cordemoyi* , *N. bourbonica* and *P. megacephala* , as only three additional species attended baits, all of which were observed in ≤ 3 experiments.

We determined the ‘winners’ and ‘losers’ from each experiment to build a dominance hierarchy. A winner was the first ant species to recruit 10 workers to the bait unless it was later usurped within the time limit. A loser was a species that was present at the bait (e.g., had found the bait or was actively foraging at it) where another species recruited 10 or more workers to it or if it was expelled by another species. Of the 85 experiments, 21 were attended by > 1 ant species and ‘winners’ and ‘losers’ were generated from this subset of the data.

### 2.4 Dietary DNA Metabarcoding

Several ant specimens were removed from each tube for identification to confirm they contained a single species. Identified reference specimens were used to confirm the identity of each ant used in dietary analysis. Ants were grasped in sterile forceps and surface‐cleaned with 1 mL of 10% bleach once and 1 mL of 100% ethanol five times. Over 90% of diet samples were of adult ants where the gaster was removed with sterile forceps and placed in a 1.5 mL centrifuge tube filled with 100% ethanol. The ant gaster contains the gut and the crop, both of which contain food. Larvae (*n* = 103) were placed in 1.5 mL centrifuge tubes filled with 100% ethanol without dissection. Adult ants typically rely on larval food transfer via trophallaxis to process and consume solid food and therefore the dietary analysis represents something closer to a colony‐level exposure to diet items, rather than individual predation events (Wulff et al. 2021). This could be in the form of liquid food ingestion, scavenging and recent prey items processed by larvae, for example. A total of 1035 ants were taken forward for DNA extraction.

Dietary metabarcoding followed Tercel et al. (2022, 2024, 2025), and detailed molecular methods can be found in Appendix S2. In brief, DNA extraction followed DNeasy Blood & Tissue Kit manufacturer recommendations, we used one negative control made of molecular grade water per seven samples, polymerase chain reactions (PCR) amplified dietary DNA in each gaster or larva using three primer pairs (two amplifying invertebrate DNA and one for plant DNA, Appendix S2, Table S3), and primers were uniquely labelled using 8 bp molecular identification tags (MID‐tags) to identify samples bioinformatically. PCR products were pooled for equimolarity and cleaned with a left‐side size selection using a 1:1 ratio. Libraries were prepared using NEXTflex Rapid DNA‐Seq Kit following the manufacturer's instructions (Bioo Scientific Corp, Austin, TX, United States) and an Agilent 4200 TapeStation with D1000 ScreenTape (Agilent Technologies, Waldbronn) to confirm ligation and fragment size. PCR products from each primer pair were sequenced separately using an Illumina MiSeq. BerenF‐LuthienR amplicons were sequenced on a V3 cartridge using 2 × 300 bp reads, and both AntExF‐AntExR and UniPlant were sequenced on separate V2 cartridges using 2 × 250 bp reads. We took forward 1241 samples with the invertebrate primers and 811 samples with UniPlant, generating an average read depth of 8151 for AntEx, 12,993 for Beren‐Lutien and 19,381 for UniPlant primers, respectively. Bioinformatics and data cleaning followed Tercel et al. (2023): FastP (Chen et al. 2018) was used to check the quality of reads, discard poor quality reads (< Q30, < 125 bp long or too many unqualified bases, denoted by ‘*N*’), trim reads to a minimum length specific to each primer pair and merge read pairs from MiSeq files (R1 and R2). Read pairs were assigned to samples and demultiplexed using Mothur v1.39.5 (Schloss et al. 2009), after which MID‐tag and primer ends were removed. Unoise3 (Edgar 2010) was used to remove replicates, denoise the sequences, and group identical sequences into zero‐radius operational taxonomic units (zOTUs, which are clustered without % identity to avoid multiple species being nested within an OTU). These zOTUs are analogous to species‐level identifications but may not be assigned a full binomial species name (i.e., family‐ or genus‐level taxonomy may be assigned if the species has not been barcoded). BLASTn was used to assign taxonomic identities to zOTUs (Camacho et al. 2009). Data were cleaned for statistical analysis broadly following the same methods as Tercel et al. (2022), whereby we removed the maximum read count found in blanks and negative controls for each taxon from all samples. After data clean‐up, 755 ant samples were taken forward for statistical analysis (Table S1). Any number of sequencing reads after data‐cleaning within a sample was considered a single detection (i.e., frequency of occurrence). Subgroup sample size for larval ants was low across five species and two seasons (*n* = 64), and therefore life stage was not statistically analysed further; adults and larvae were analysed together indiscriminately.

### 2.5 Statistical Analyses

#### 2.5.1 Is Intraguild Predation Frequent?

All statistical analyses were conducted in R version 4.3.1 (R Core Team 2023). To gauge the importance of ant‐ant trophic interactions for the community, we calculated basic dietary statistics for each species and the proportion of the diet made up of other ant species. We tested whether diet differed among ant species using R package ‘mvabund’ (Wang et al. 2012). Multivariate generalised linear models (MGLMs) were run using the ‘manyglm’ function with a Monte Carlo resampling method and binomial error family. We tested whether the frequency of intraguild predation deviated from the level expected based on the overall abundance of ants in the arthropod community by using null network models in R package ‘econullnetr’ with 1000 simulations (Vaughan et al. 2018). This was done by comparing the diet of individual ants in each quadrat to the abundance of arthropod taxa found in the corresponding pitfall traps. For this analysis we pooled all dietary and community taxa into ‘ants’ and ‘non‐ants’ and generated null networks from those data. Furthermore, we conducted an identical analysis with *P. megacephala* excluded because of its abundance and behavioural dominance, which we believed may influence the community disproportionately when pooled with other ant species. Consumption frequencies that fell outside the 95% confidence limits generated by the null model highlighted interactions that were stronger or weaker than expected, suggesting preference for, or avoidance of, particular prey species. To aid comparisons among species, preferences were expressed as standardised effect sizes, where negative values represent less frequent interactions than expected, positive values more frequent and zero indicates that the consumption frequency was the same as predicted by the null model (Vaughan et al. 2018).

#### 2.5.2 Are Intraguild Predation Patterns Non‐Random?

To test whether some ants avoid others or interact as expected by chance we used two different analyses. Firstly, we calculated the extent to which the co‐occurrence of ant species in quadrats differed from random based on hand collection data in each of the 69 quadrats sampled across Round Island using R package ‘cooccur’ (Griffith et al. 2016). We conducted co‐occurrence analyses separately for wet and dry seasons. We then used the econullnetr package to test whether ant consumers ate other ant species (non‐ant prey was excluded) as frequently as expected based on their abundance or disproportionately more/less frequently: the null models compared the dietary data of individual ants in each quadrat to the abundance of other ant species found in pitfall traps of the associated quadrat.

#### 2.5.3 Do Behaviourally Dominant Ants Influence the Access to Food for Intraguild Species?

Limits to inference from cooccurrence analysis are well known (Blanchet et al. 2020), so we strengthened our ability to infer why non‐random cooccurrence patterns might arise by obtaining direct behavioural observations to see whether behavioural interactions were consistent with statistical co‐occurrence patterns. We did this by quantifying how quickly the three commonest ant species on Round Island ( *B. cordemoyi* , *N. bourbonica* and *P. megacephala* ) found and recruited to baits, the proportion of baits attended, the average number of other ant species present at baits with each species, and antagonistic behaviours and displacement arising from these. Data were not normally distributed, so Wilcoxon rank sum tests were used to test for differences in the ability of the three species to find and recruit to baits. We built a behavioural dominance hierarchy with the Elo ranking system commonly applied to professional competitions (e.g., chess and online gaming), which has also been used in biological contexts (Yitbarek and Philpott 2019). Elo scores were calculated from the winners and losers of each experiment in R package ‘aniDom’ (Farine and Sanchez‐Tojar 2022) using the ‘elo_scores’ function over 1000 randomly ordered interaction outcomes (‘n.rands = 1000’) for each species. We estimated the level of uncertainty in the final dominance rank for the three ant species using the function ‘estimate_uncertainty_by_splitting’ across 1000 randomly ordered interaction outcomes.

#### 2.5.4 Do Behaviourally Dominant Ants Engage in More Competitive Interactions?

Given that ant species' competitiveness is likely to be linked to their dominance, we quantified potential competition between each species pair based on their second order interactions (i.e., resource sharing). The frequency and identity of these predator–prey–predator interactions, where ants share (ant and non‐ant) prey at the same points in space and time, provided a simple proxy for intraguild competition for resources. The frequency of interactions between ant and prey species was first calculated for each quadrat and season. From this, the frequency of indirect (second order) ant‐ant interactions was calculated, in which two ants were considered indirectly interacting if those species shared a prey species within the same quadrat (independent of direct interactions between ants), and the frequency was considered the number of shared interactions; for example, if Ant A consumed Prey B 13 times, and Ant C consumed Prey B 34 times, the frequency of indirect interaction in this instance was considered 13. These interactions were used to construct hypergraphs (i.e., networks in which links join more than two nodes) for each quadrat, in this case linking pairs of predators and their shared prey. These hypergraphs were then pooled to create overall hypergraphs for the two seasons. The second‐order intraguild interactions from these hypergraphs were visualised using conventional network plots, constructed using the ‘igraph’, ‘ggplot2’, ‘ggnetwork’ and ‘scatterpie’ packages (Csardi and Nepusz 2006; Wickham 2016; Briatte 2021; Yu 2021).

Competitive interaction richness, defined as the number of prey species featuring in competitive interactions between each ant pair, and frequency, the total number of competitive interactions, were compared between ant species and seasons in generalised linear mixed‐effects models (GLMMs) using the ‘glmer’ function in the ‘lme4’ R package (Bates 2015), with the quadrat number included as a random factor. Both the richness and frequency GLMMs used a Poisson error family and model assumptions were checked using the ‘DHARMa’ package (Hartig 2022).

## 3 Results

### 3.1 Intraguild Trophic Interactions Are Disproportionately Frequent

**FIGURE 2:**
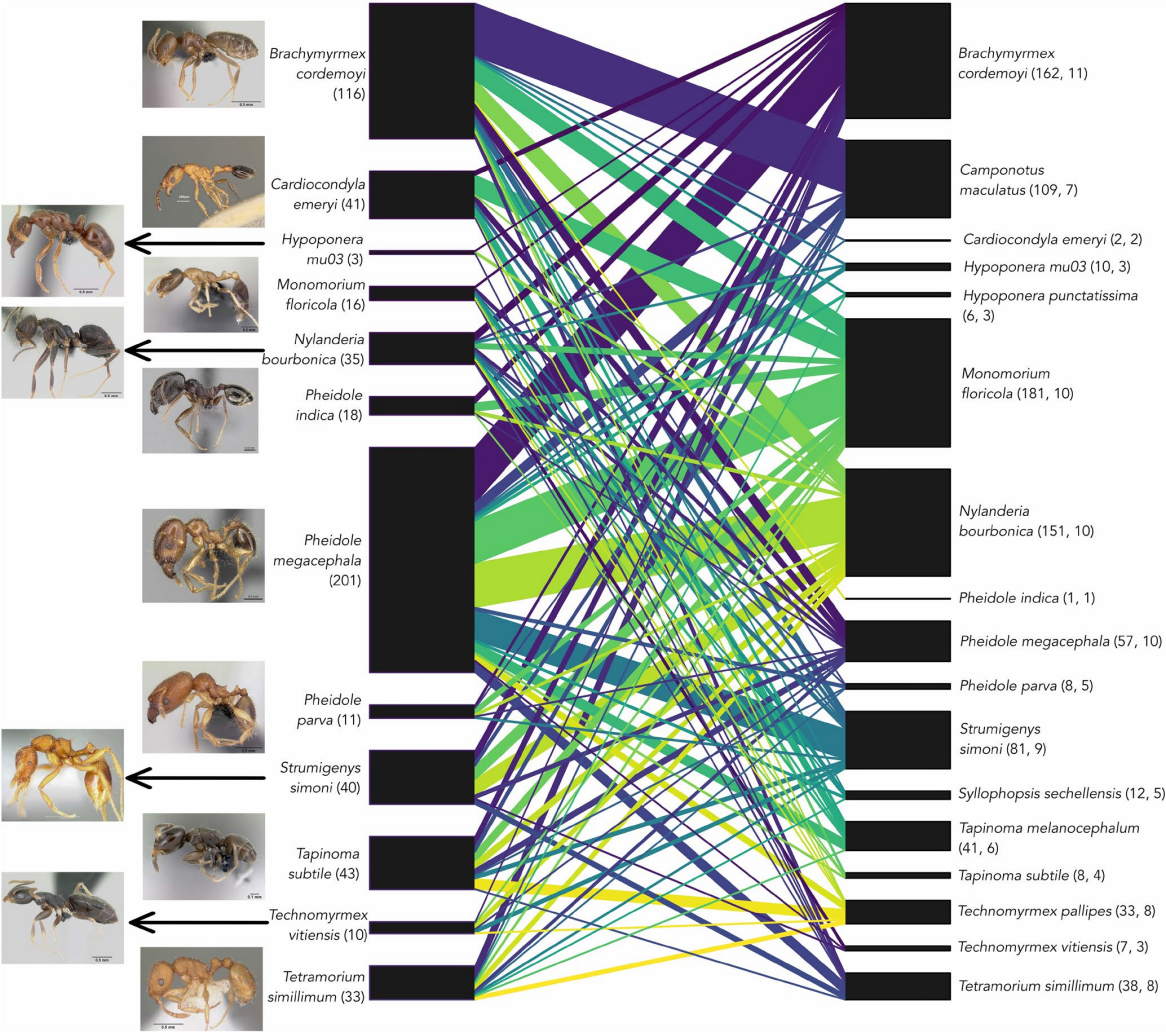
Intraguild trophic interactions showing consumers (left) and dietary taxa (right). The height of black rectangles, and the width of coloured links between them, is proportional to the number of detections. Links are arbitrarily coloured to aid visualisation. Numbers in parentheses beside consumer names indicate the sample size. Numbers in parentheses beside dietary taxa indicate the number of detections and how many species it was consumed by, respectively. Photos downloaded from AntWeb.org v8.106.1 under CC‐BY 4.0; see Appendix S3 for photo attributions.

**FIGURE 3:**
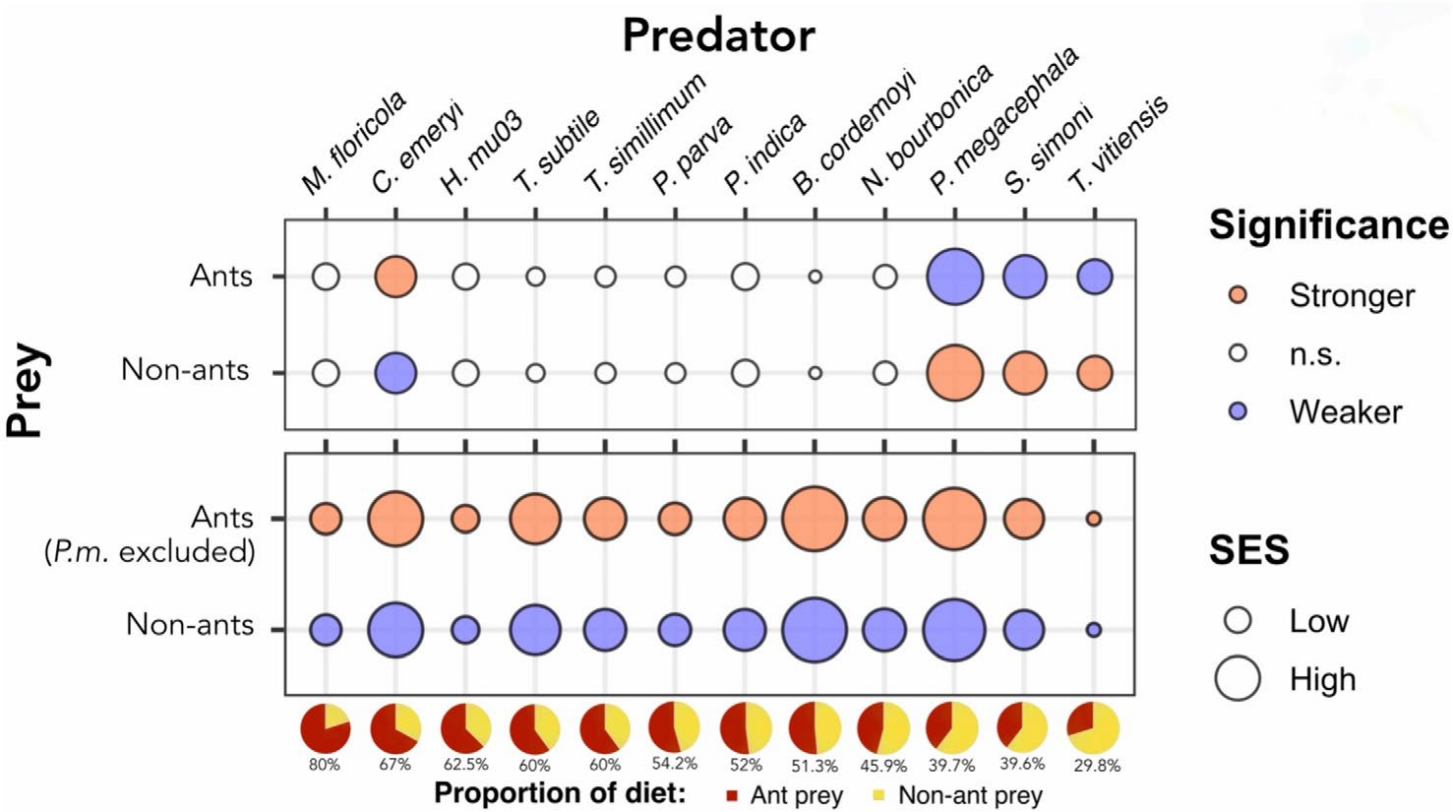
Prey choice standardised effect sizes (SESs) are given between prey resources (‘ants’ and ‘non‐ants’) and predator species. Larger points reflect larger SESs (zero indicating the absence of preference). Orange, white and blue points show stronger (preference), non‐significant (density‐dependent) and weaker (avoidance) interactions compared to the null model based on 95% confidence intervals. Predator columns are in descending order from left to right by the proportion of ant prey in the diet. The upper plot shows all potential ant prey pooled together. The lower plot shows potential ant prey pooled together with *Pheidole megacephala* excluded. Pie charts at the base of the plot denote the proportion of ant and non‐ant prey in the diets of each predator species, and associated percentages equal how much of the total diet is made up of ant prey.

Dietary analysis detected 1952 trophic interactions with 158 dietary taxa from 755 ants belonging to 12 non‐native species (Figure 2). Ant‐ant trophic interactions comprised 46.5% of dietary detections, with the mean proportion of intraguild consumption across the 12 consumer species being 53.5% (± 13.1 SD; range = 29.8%–80%; Figure 3). Null models indicated that the frequency of intraguild predation was usually proportional to the abundance of ant prey (Figure 3, upper plot), indicating neither preference for or avoidance of intraguild prey: while one species consumed ants more frequently, and three species less frequently, eight species (67%) consumed them as expected based on their abundance. However, when *P. megacephala* were removed as a potential prey resource before pooling, all predators consumed intraguild prey significantly more than expected based on its abundance (Figure 3, lower plot).

Dietary composition varied with season (LRT = 539, *p* < 0.001) and separate analyses were therefore conducted using wet and dry season data. Ant‐ant dietary composition varied significantly between consumer species in both the wet and dry seasons (wet: LRT = 440.9, *p* < 0.001; dry: LRT = 279.4, *p* = 0.001; Appendix S4, Figure S4), suggesting partitioning between species' intraguild diets.

### 3.2 Intraguild Predation Patterns Are Non‐Random

The co‐occurrence analysis revealed 11 random and two negative co‐occurrence relationships. Both negative co‐occurrence relationships existed with *P. megacephala* , one each with *N. bourbonica* and *Pheidole indica* .

**FIGURE 4:**
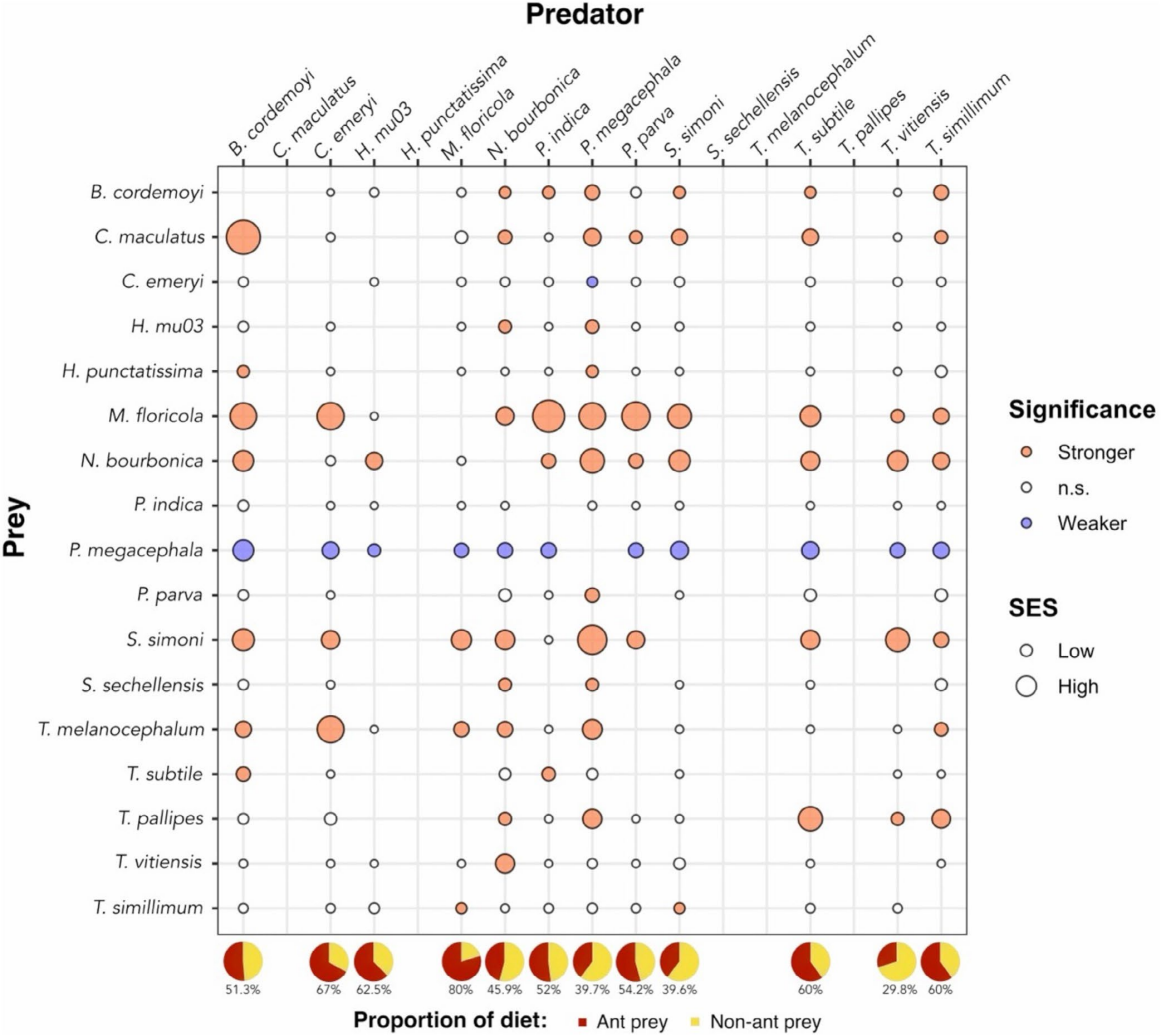
Prey choice standardised effect sizes (SESs) are given between ant predators and prey. Larger points reflect larger absolute SES values (zero indicating the absence of preference). Orange, white and blue points show stronger (preference), non‐significant (density‐dependent) and weaker (avoidance) interactions compared to the null model based on 95% confidence intervals. Absent points are where data were not available or are same‐species pairs. Pie charts at the base of the plot denote the proportion of ant and non‐ant prey in the diets of each predator species, and associated percentages equal how much of the total diet is made up of ant prey.

Prey choice analysis identified a complex series of relationships in the ant community (Figure 4), including density‐dependent consumption and disproportionate avoidance/selection of certain prey. There were numerous species‐specific prey preferences revealed by the analysis, but most notably all ants consumed *P. megacephala* less than expected, while *Monomorium floricola* , *Strumigenys simoni* and *N. bourbonica* were almost always predated more often than expected (in 10/11, 9/10 and 9/11 of interactions, respectively). The ant community consumed *Cardiocondyla emeryi* as expected based on its abundance with the exception of *P. megacephala* , which avoided it. A substantial portion of potential pairwise interactions between ant species pairs could not be measured because those species did not co‐occur.

### 3.3 Behaviourally Dominant Species Influence Food Availability for Intraguild Species

**FIGURE 5:**
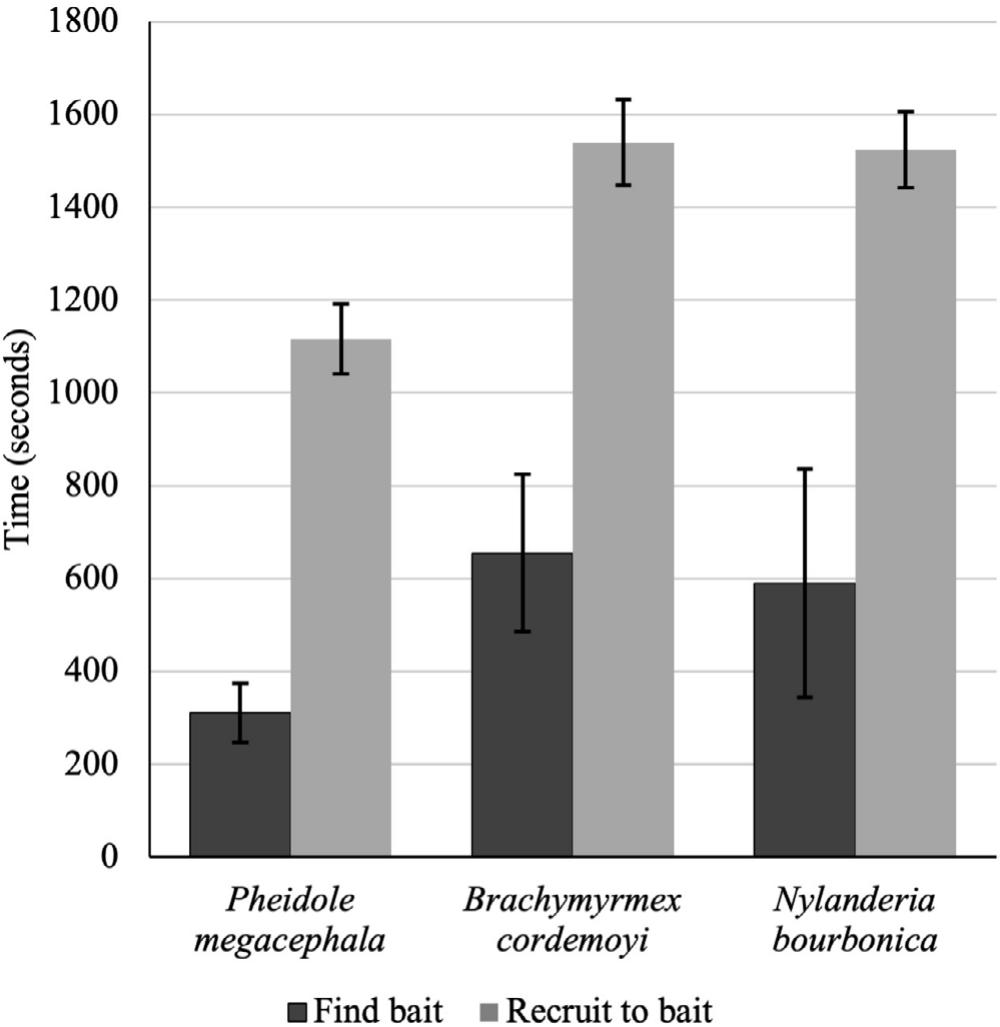
Mean (± SE) time taken for ant workers to find (dark grey) and recruit (light grey) to baits between the three focal species.

*Pheidole megacephala* found and recruited to baits significantly faster than both *B. cordemoyi* (Wilcoxon Rank Sum tests: finding: *W* = 236.5, *p* = 0.004; recruiting: *W* = 301.5, *p* = 0.034; Table 1) and *N. bourbonica* (finding: W = 168, *p* = 0.028; recruiting: *W* = 181, *p* = 0.042); we found no significant difference in the speed at which *B. cordemoyi* and *N. bourbonica* found or recruited to baits (finding: *W* = 70, *p* = 0.680; recruiting: *W* = 64.5, *p* = 0.550) (Figure 5). Moreover, we found that *P. megacephala* co‐occurred at baits with significantly fewer other ant species than both *B. cordemoyi* and *N. bourbonica* ( *B. cordemoyi* : *W* = 52, *p* < 0.0001; *N. bourbonica*: *W* = 36.5, *p* < 0.0001; Table 1) and similarly found no significant difference between the latter pair (*W* = 67.5, *p* = 0.640).

**TABLE 1:** Measures of bait discovery and control ability of the three most abundant ant species on Round Island based on 85 thirty‐min island‐wide baiting experiments.

Discovery‐dominance measurement	*Brachymyrmex cordemoyi*	*Nylanderia bourbonica*	*Pheidole megacephala*
% of baits present (*n* = 85)	28.24	18.82	90.59
% of baits present where workers were first to find bait	58.33	56.25	80.52
% of baits found first where 10 workers were subsequently recruited within 30 min	55.56	14.29	90.32
% of baits present where this species was first to recruit 10 workers	8.33	50.00	79.22
% of baits present where this species expelled a faster coloniser	0.00	42.86	33.33
% of baits expelled by another species	42.86	33.33	0.00
Average number of cooccurring ant species (± SE)	1.58 (± 0.15)	1.63 (± 0.18)	0.43 (± 0.08)

**FIGURE 6:**
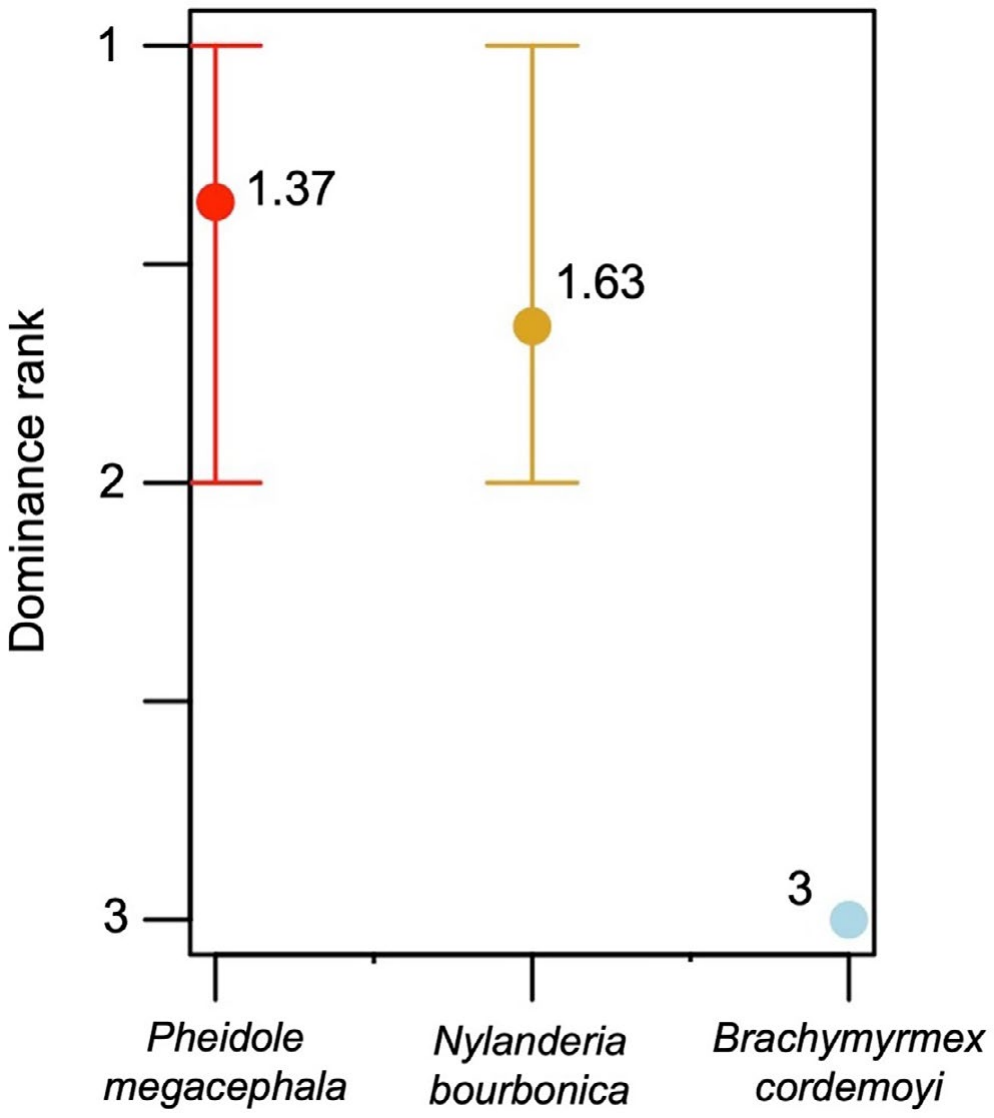
Behavioural dominance ranks calculated from Elo scores (±95% CI) from interaction outcomes at 21 thirty‐min baiting experiments for the three most abundant ants on Round Island, *B. cordemoyi* , *N. bourbonica* and *P. megacephala* .

*Pheidole megacephala* and *N. bourbonica* displayed similar behavioural dominance, with the former slightly more dominant on average (Figure 6). Of the three species tested, *B. cordemoyi* had a significantly lower dominance score, failing to expel either of the other species from any of the baits at which it was present. No ties between species occurred. Our uncertainty analysis showed that results were very similar between the two halves of the dataset and, overall, we found a high level of reliability in our Elo scoring analysis (Spearman's rank correlation: mean = 0.74, CI = ±0.25).

### 3.4 Ant Species Differ in the Frequency of Their Competitive Interactions

**FIGURE 7:**
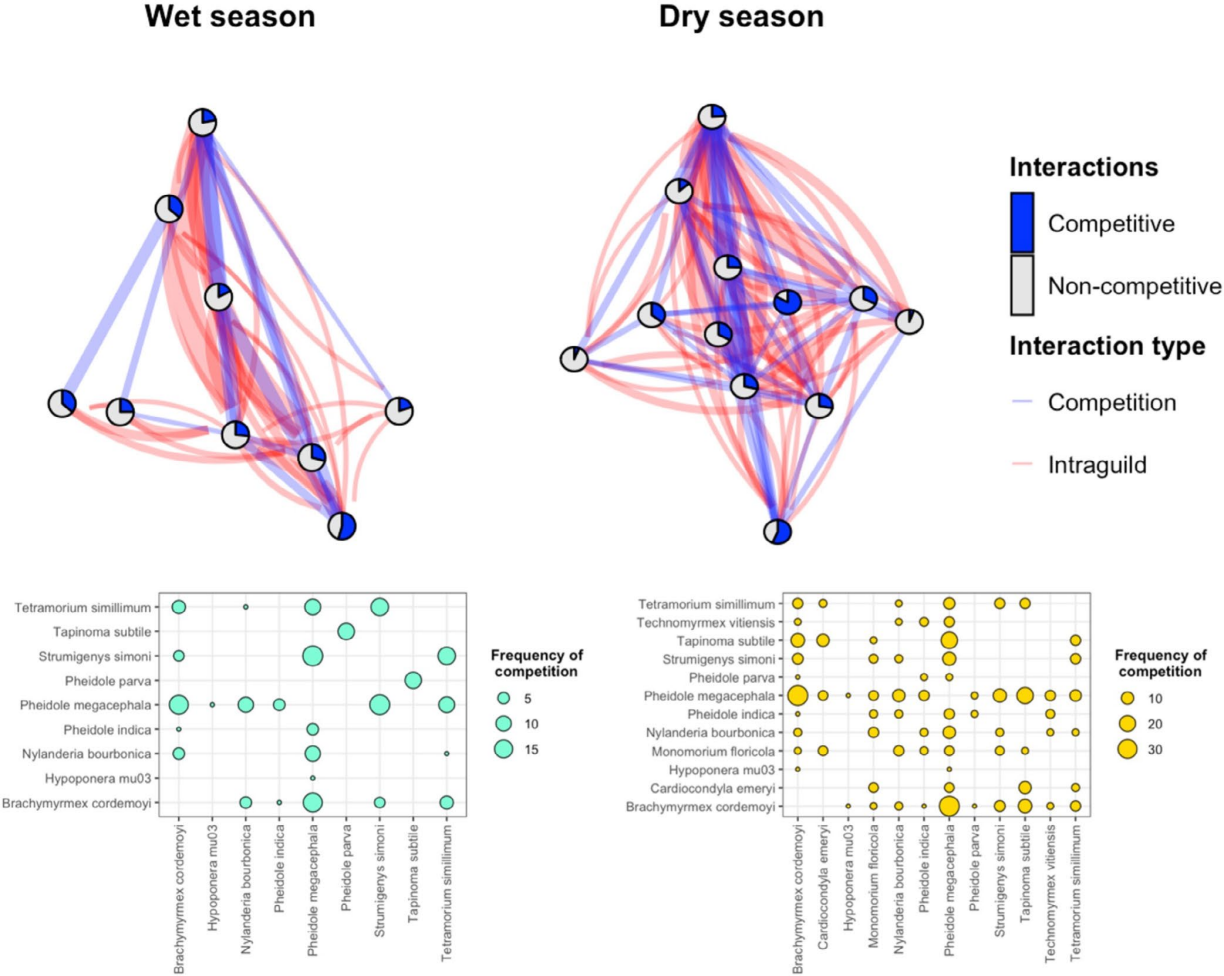
Networks of direct links between ants, including direct ant‐ant (intraguild; curved red links), and indirect second order ant‐ant (competition; straight blue links) interactions. Pie charts represent ant nodes, with the ratio of grey to blue denoting the proportion of non‐competitive (i.e., prey not shared with another ant in the same space and time) to competitive (i.e., prey shared with another ant in the same space and time) interactions with prey, respectively. The plots below show the frequency of competitive interactions between different ant species. Plots to the left are based on wet season data, and the right represent the dry season. The prey for which ants were competing are represented in Appendix S4, Figure S5. Networks including prey nodes are presented in Appendix S4, Figure S6.

**FIGURE 8:**
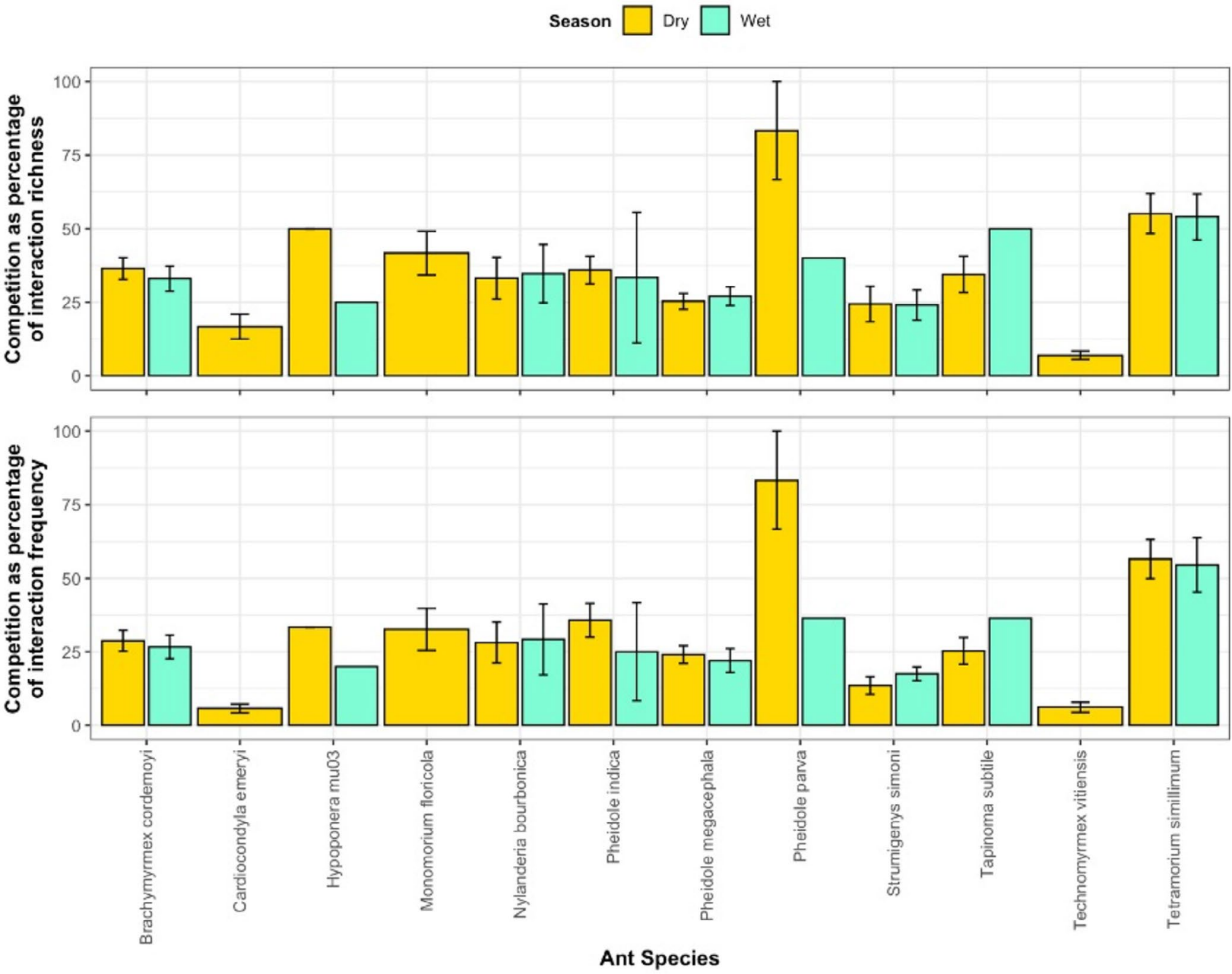
The percentage of ant‐prey interactions that were shared with other ants in the same space and time for each ant across each season, expressed as a percentage of interaction richness (top) and frequency (bottom).

The richness of competitive interactions (i.e., the number of prey species shared by pairs of ants) did not differ significantly between seasons (Figure 7; Appendix S5, Figure S5), but did differ significantly between some ant species (Appendix S5, Table S4). The frequencies of competitive interactions were significantly higher in the dry season (GLMM: estimate = 0.190 ± 0.087, *z* = 2.186, *p* = 0.029; Figure 7; Appendix S5, Figure S6) and differed significantly between some ant species (Appendix S5, Table S5). Similar results were obtained if competitive interaction richness and frequency were converted to proportions of the total interactions, controlling for differences in prey abundance, except that seasonal differences were no longer significant (Appendix S5, Tables S6 and S7; Figure 8).

## 4 Discussion

Our results indicate that intraguild interactions are central to the dynamics of a non‐native community. This advances our conceptual understanding of intraguild interactions from native communities into invaded communities based on our three central hypotheses: (1) Intraguild interactions were widespread and important, comprising roughly half of prey detections, while one third of the prey detected represented potential exploitation competition between ants. Each ant species frequently interacted as predator, prey, or both. Moreover, null models suggested that with the notable exception of *P. megacephala* as a prey resource, intraguild interactions were much more frequent than expected based on the abundance of ant and non‐ant prey. (2) Intraguild trophic interactions were non‐random, with strong evidence for prey preferences. In addition, several ant species avoided areas with *P. megacephala* , consistent with their subordinate position to *P. megacephala* in our behavioural experiments, suggesting that dominant intraguild species can spatially structure non‐native communities. Despite this, the potential for competition remained high, with 29.5% of ant trophic interactions representing likely competition since the same prey were shared by multiple ants at the same locality. (3) Ecologically dominant species restrict space use and food resource access of other non‐native ants, contextually structuring the activity and behaviour of other intraguild species at small spatial scales.

### 4.1 Hypothesis 1: Intraguild Predation Is Prevalent and Ecologically Important

Our finding that roughly half of trophic interactions involved intraguild consumption is comparable to, or even exceeds, intraguild predation frequency documented in native systems (Arim and Marquet 2004) and aligns with theoretical expectations for communities structured by intraguild interactions (Polis et al. 1989). The importance of intraguild predation to the overall diet for each of our consumer species varied substantially, from 80% of the diet in *M. floricola* to 30% in *T. vitiensis* .

These results suggest that intraguild predation is a fundamental foraging strategy among non‐native ants. While dietary metabarcoding cannot reveal the biomass consumed or nutrition obtained by predators (Lamb et al. 2019), the high incidence of ant prey suggests that consumption of guild members is common and ecologically relevant. The exact contribution to diet is likely to vary according to consumers' life history traits, foraging behaviour, and resource use strategies. For example, the high frequency of ant consumption by the small, slow‐moving species *M. floricola* may arise from scavenging (Wetterer 2010), rather than intraguild predation *sensu stricto*. Conversely, *P. megacephala* , despite being known to raid other ant nests (Dejean et al. 2008), showed low overall ant consumption, probably as a result of its ability to access and control other food resources. *P. megacephala* consumed most ant species more than expected based on their abundance, but other species were found infrequently in areas with *P. megacephala* , which may reflect historical intraguild predation near nests. Many other predators have been shown to disproportionately exclude intraguild species within their territories or around their nests (Polis et al. 1989; Björklund et al. 2016). The proportion of the diet made up of intraguild species is therefore not solely based on the predation effectiveness of a consumer, but also the historical priority effects of local colonisation (Polis et al. 1989), the ability of a species to exploit different strategies of consumption, and the abundance of respective prey species. Overall, these findings support the idea that intraguild consumption is influenced by species‐specific traits and foraging flexibility.

The distinct trophic ecology of ants and the varied foraging modes they use presents a unique challenge for ecologists aiming to understand their diet with high taxonomic resolution. Because of the way that ants feed and share food resources through trophallaxis, each individual sample represents something closer to a colony‐level exposure to the detected food resources rather than individual‐level predation events. Ant diet samples are therefore probably close to a theoretical ‘average’ colony‐level diet composition because of multiple food exchanges between workers that bring back solid food items and larvae that consume, digest and regurgitate some of the same food items back for any worker to consume through trophallactic transfer. This is sometimes called the ‘social stomach’ (Hölldobler and Wilson 2009) because larval consumption and regurgitation allow large solid food items to be broken down and shared with other members of the colony. Furthermore, the varied foraging strategies of ants provide another challenge for dietary metabarcoding because food items in our study may have been collected through predation or scavenging, as is common in many non‐native ants (Holway and Cameron 2021). Scavenging is especially important to the detrital brown food web, which non‐native ants frequently exploit (Holway and Cameron 2021), and neglecting this important mode of foraging could underestimate the importance of ants to the wider soil community. Dietary detections of animal food resources are therefore probably a mixture of scavenging and predation.

Our results also highlight the complexity of intraguild consumption among ants, which may occur through either scavenging or predation. While dietary metabarcoding cannot distinguish between these modes, both behaviours are plausible given that ants rapidly monopolise carrion and may scavenge dead ants discarded from other nests. Species‐specific traits likely influence whether intraguild interactions involve active predation or the removal of dying or dead individuals, and further observational studies at nest sites could clarify these dynamics.

### 4.2 Hypothesis 2: Intraguild Interactions Are Non‐Random and Species‐Specific

We found strong evidence that intraguild consumption is non‐random, instead being structured by prey identity, species‐specific preferences, and local spatial co‐occurrence. All consumer species we studied showed some non‐random patterns of intraguild predation. Some species were consumed disproportionately by most other ant species (e.g., *M. floricola* , *S. simoni* , *N. bourbonica*). In contrast, *P. megacephala* was avoided by all consumers. The outcomes of intraguild interactions may be context dependent; temperature (Hölldobler and Wilson 1990), group size (Franks and Partridge 1993), distance from the nest (Lach et al. 2010), the brood cycle of the colony (Franks and Fletcher 1983), and combat prowess (Hölldobler and Wilson 1990; Franks and Partridge 1993) may all determine which ant species consumes another in a given situation. An analysis of how these varied traits influence intraguild interactions is outside the remit of this study but is an avenue for future research in the context of non‐native communities.

Our co‐occurrence analysis further revealed that two species (*N. bourbonica* and *P. indica* ) co‐occur with *P. megacephala* less frequently across the island than expected. *Pheidole megacephala* is thought to be one of the world's worst invasive species because of its impact on native species (Hoffmann and Parr 2008), and our results suggest it can exert a substantial structuring force on ant communities even when they consist entirely of other non‐native species. While co‐occurrence data alone do not necessarily provide strong evidence that two species are interacting (Blanchet et al. 2020), results from the food bait trials (discussed further below) support the conclusion that the negative co‐occurrences between *P. megacephala* and other species likely arise because of behavioural exclusion/avoidance. Taken together, these results suggest that spatial partitioning may emerge as an outcome of intraguild pressure, especially where dominant species regulate both space and access to food. Non‐random co‐occurrence is consistent with theoretical models predicting that intraguild predators influence community composition through spatial exclusion (Polis et al. 1989; Björklund et al. 2016). Furthermore, our findings reinforce the idea that non‐native communities self‐organise as a result of interaction‐based hierarchies, much like native ant communities (Vepsalainen and Savolainen 1990; Parr et al. 2005), rather than random assemblages of co‐introduced species.

### 4.3 Hypothesis 3: Ecological Dominance Structures Food Access and Local Activity Patterns

Experimental food baits and competitive interaction networks showed that ecological dominance can restrict access to resources for other ants, influencing local activity patterns and spatial cooccurrence, although the food bait analysis was limited to the three most abundant species and lacked coverage for many other species pairs. When paired with diet and spatial data, results from the behavioural trials support the conclusion that *P. megacephala* is a dominant species with guild‐wide effects. While large food baits are imperfect representations of food resources in the environment (Lach et al. 2010), they can provide useful data describing the speed at which species find and defend food resources from competitors and are widely used in ant ecology. *Pheidole megacephala* found and recruited to baits more quickly than other species, was never expelled, and co‐occurred with fewer competitors at food sources—demonstrating a high food discovery ability and behavioural dominance. This challenges the classic dominance‐discovery trade‐off posited in ant ecology (Hölldobler and Wilson 1990), where species are placed along a spectrum of either good discoverers or defenders of food resources, because *P. megacephala* excels at both. This trade‐off has been challenged before in invasive species contexts (Arnan et al. 2018) and the great abundance of *P. megacephala* across Round Island might be partly explained by its release from this posited trade‐off, probably resulting from an absence of similarly competitive species. Intraguild species likely coexist by avoiding *P. megacephala* where possible, as observed in most of the food baits with *B. cordemoyi* and *N. bourbonica*, or specific adaptations that allow them to co‐exist alongside *P. megacephala* . *Cardiocondyla emeryi* , for example, was the only species *P. megacephala* consumed less than expected based on its abundance and emits a repellent pheromone that enables it to coexist with dominant species elsewhere in its native and introduced range (Creighton and Snelling 1974).

We further observed that *P. megacephala* and *B. cordemoyi* competed frequently in second‐order interactions, that is, potential exploitation competition through shared prey. We observed these indirect interactions with multiple ant species across both seasons, aligning with their relative dominance of resources. These interactions may constrain the resource base available to less dominant species and reinforce exclusion through exploitation competition. *Nylanderia bourbonica*, which was the second most dominant ant in the baiting experiments, sparsely engaged in competitive interactions with other ants in the wet season, and only moderately in the dry season. It was often found on vegetation, unlike many other ants, and thus may have avoided competition via spatial segregation. Given *N. bourbonica*'s high dominance, it is perhaps more likely that other ants avoided resources that it colonised, or that it was able to extirpate competitive ants. Importantly, *P. megacephala* is the only ant with a higher dominance score and yet engaged frequently in competitive interactions; this may simply be due to *P. megacephala* 's vast abundance, accounting for 85% of the invertebrates caught in pitfall traps. This abundance may mean that other ants are forced to co‐occur and compete with *P. megacephala* unless they can access alternative strata (e.g., tree canopies). *Nylanderia bourbonica* and *P. megacephala* may therefore be similarly deleterious to other ants, but *N. bourbonica*'s lower abundance simply masks this in the context of competition. When comparing their competitive interactions as percentages of total interactions, *P. megacephala* and *N. bourbonica* had similar rates of competition (and similar again to *B. cordemoyi* ), supporting the idea that they could have similar impacts if equally abundant.

A major piece of evidence enabling us to make grounded ecological inferences came from the food bait trials and their complementarity with spatial cooccurrence and diet data. Though the behavioural bait trials cover the three most abundant species on Round Island, together comprising ~90% of ant abundance, a greater coverage of the other possible species pairs in the community would improve the resolution of this analysis further. Nevertheless, a holistic synthesis of these different threads of evidence suggests that the ant community on Round Island is dominated by *P. megacephala* , but species‐specific trait‐mediated trade‐offs allow for coexistence with other ants, possibly at the cost to resource breadth, foraging efficiency, or optimal nesting location.

## 5 Conclusions

In summary, our results provide strong evidence that intraguild interactions are central to the structure and dynamics of non‐native communities, even in the absence of shared evolutionary history. In a fully non‐native community, intraguild interactions influenced diet, the local distribution of species, and competition at food resources—ultimately governing community composition and behaviour. In line with long‐standing observations in native systems, we argue that ant‐ant interactions are a ‘hallmark of ant ecology’ (Hölldobler and Wilson 1990) regardless of origin.

More broadly, our results have important implications for understanding the assembly of non‐native communities and their wider ecological effects. For example, intraguild interactions might mediate the net impact of invasive species on native biota. In some contexts, less damaging non‐native species may buffer against more harmful invaders by occupying space and using resources, which has been previously investigated in crabs (Jensen et al. 2007), mussels (Ricciardi 2003), plants (Kuebbing and Nuñez 2015) and ants (Holway et al. 2002), but such interactions are generally poorly quantified. Understanding these and other intraguild processes will be critical for predicting and managing the dynamics of increasingly common multi‐invader ecosystems.

## Author Contributions

Maximillian P.T.G. Tercel, Nik C. Cole, William O.C. Symondson, and Ian P. Vaughan designed the study. Maximillian P.T.G. Tercel collected data. Maximillian P.T.G. Tercel, Ian P. Vaughan, and Jordan P. Cuff analysed the data. Maximillian P.T.G. Tercel led the writing of the manuscript. William O.C. Symondson, Nik C. Cole, Martine Goder, Kevin Ruhomaun, and Vikash Tatayah assisted Maximillian P.T.G. Tercel with logistics and sample collection in the field. All authors significantly contributed to the critical appraisal of the manuscript.

## Funding

Maximillian P.T.G. Tercel was funded by the Durrell Wildlife Conservation Trust (MR/S502455/1) and the Natural Environment Research Council (NE/L002434/1). Jordan P. Cuff was funded by a Newcastle University Academic Track Fellowship.

## Disclosure

Benefit Sharing Statement: Benefits generated: this research represents a collaboration between scientists in Mauritius and the UK, with both groups benefiting through two‐way knowledge transfer and exchange. All collaborators are included as co‐authors. The results of the research have been shared with communities in both countries and the broader scientific community.

## Ethics Statement

The authors declare that all laws and permissions were followed and obtained, respectively, in order to conduct the current study.

## Conflicts of Interest

The authors declare no conflicts of interest.

## Supporting information


**Figure S1:** Skirted canvas pitfall trap suitable for rocky substrates.
**Figure S2:** Abundance of invertebrates captured in 77 pitfall traps over Round Island. Ants are given lower taxonomic information, while other taxa were identified to order or class. Note the logarithmic scale.
**Figure S3:** Results of *in silico* amplification testing using PrimerMiner to determine the amplification efficiency of AntEx for a range of different taxa present on Round Island.
**Figure S4:** Ant species diet composition of other ants consumed between wet (a) and dry (b) seasons visualised using non‐metric multidimensional scaling (NMDS). Colours denote different species of ant consumers, and the large points show the associated mean dietary composition of a given species and are annotated with binomial species names beside each; terminal ends of lines represent the dietary composition of individual ants and are connected to their associated centroid.
**Figure S5:** The frequency with which ants were competing for different prey. Frequencies denote the number of occurrences of each prey taxon in competitive interactions between ants, with yellow representing the dry season and green the wet season.
**Figure S6:** Networks of direct ant‐non‐ant‐prey (trophic; curved grey links) and ant‐ant (intraguild; curved red links), and indirect second order ant‐ant (competition; straight blue links) interactions. Pie charts represent ant nodes, with the ratio of grey to blue denoting the proportion of non‐competitive (i.e., prey not shared with another ant in the same space and time) to competitive (i.e., prey shared with another ant in the same space and time) interactions with prey, respectively. The plots below show the frequency of competitive interactions between different ant species. Plots to the left are based on wet season data, and the right represent the dry season.
**Table S1:** Ant species recorded on Round Island with life history information and their use in this study; for the ‘diet analysis’ column, numbers in parentheses indicate the number of samples with diet data after data cleaning.
**Table S2:** The ecological principles of interest and scales at which they are examined in the present study, including replicates for each factor.
**Table S3:** Primers used in the current study. AntEx primers were designed for the current study, targeting a 214 bp amplicon of the mitochondrial COI gene. BerenF‐LuthienR and UniPlant primers amplify 314 bp and 250 bp amplicons from the COI and ITS2 markers, respectively.
**Table S4:** Comparison of the competitive richness of each ant taxon across the study. Values are the estimate, standard error, *z*‐value and *p*‐value of a GLMM.
**Table S5:** Comparison of the frequency of competitive interactions that each ant taxon engaged in across the study. Values are the estimate, standard error, *z*‐value and *p*‐value of a GLMM.
**Table S6:** Comparison of the percentage the richness of ant‐prey interactions that were shared with other ants in the same space and time between each ant taxon across the study. Values are the estimate, standard error, *t*‐value and *p*‐value of a LMM.
**Table S7:** Comparison of the percentage of the frequency of ant‐prey interactions that were shared with other ants in the same space and time between each ant taxon across the study. Values are the estimate, standard error, *t*‐value and *p*‐value of a LMM.
**Appendix S1:** Ant community and invertebrate sampling.
**Appendix S2:** Detailed molecular pipeline and PCR primers.
**Appendix S3:** Photo attributions for Figure 2
**Appendix S4:** Diet composition and NMDS plots.
**Appendix S5:** Competitive interactions.

## Data Availability

All code and data files are publicly available at https://zenodo.org (10.5281/zenodo.15483834).
