# Supplementary material for: Intraguild Interactions Drive the Dynamics of a Complex Community of Globally Invasive Ant Species
Source: Mol Ecol. 2026 Jul 9;35(13):e70464. doi: 10.1111/mec.70464 (PMC13350404; doi:10.1111/mec.70464)
Supplement: Supplementary file 1 — Figure S1: Skirted canvas pitfall trap suitable for rocky substrates. Figure S2: Abundance of invertebrates captured in 77 pitfall traps over Round Island. Ants are given lower taxonomic information, while other taxa were identified to order or class. Note the logarithmic scale. Figure S3: Results of in silico amplification testing using PrimerMiner to determine the amplification efficiency of AntEx for a range of different taxa present on Round Island. Figure S4: Ant species diet composition of other ants consumed between wet (a) and dry (b) seasons visualised using non‐metric multidimensional scaling (NMDS). Colours denote different species of ant consumers, and the large points show the associated mean dietary composition of a given species and are annotated with binomial species names beside each; terminal ends of lines represent the dietary composition of individual ants and are connected to their associated centroid. Figure S5: The frequency with which ants were competing for different prey. Frequencies denote the number of occurrences of each prey taxon in competitive interactions between ants, with yellow representing the dry season and green the wet season. Figure S6: Networks of direct ant‐non‐ant‐prey (trophic; curved grey links) and ant‐ant (intraguild; curved red links), and indirect second order ant‐ant (competition; straight blue links) interactions. Pie charts represent ant nodes, with the ratio of grey to blue denoting the proportion of non‐competitive (i.e., prey not shared with another ant in the same space and time) to competitive (i.e., prey shared with another ant in the same space and time) interactions with prey, respectively. The plots below show the frequency of competitive interactions between different ant species. Plots to the left are based on wet season data, and the right represent the dry season. Table S1: Ant species recorded on Round Island with life history information and their use in this study; for the ‘diet analysis’ column, n [file MEC-35-e70464-s001.docx]

**Appendix**

Manuscript title: *Intraguild interactions drive community dynamics in invasive ants*

Journal: *Journal of Animal Ecology*

Appendix S1. Ant community and invertebrate sampling.

There are 18 ant species recorded on Round Island (Table S1), including new records from this study. Based on records from mainland Mauritius dating back to the late 18^th^ century, it is possible some of the Mauritian islets were colonised by non-native ants soon after Mauritius was visited by European sailors. For example, the holotype for the African big-headed ant *Pheidole megacephala* was collected in Mauritius (Fabricius 1793). Because of this, it was originally thought that *P. megacephala* was native to Mauritius, but unidentified specimens collected many years earlier suggest it originates either from mainland Africa or Madagascar (Wetterer 2012). Similarly, *Nylanderia bourbonica* was originally described from a specimen collected in Réunion, but is native to South-East Asia (Deyrup 2017) and *Tetramorium simillimum* and *Camponotus maculatus* have been present on Round Island since at least 1975 (collected by D. Bullock) and 2001 (observed by N. Cole and V. Tatayah), respectively. It is also not known whether Round Island ever hosted native ants because no records of ants exist before the degradation of the island’s native forests. The loss of topsoil and vegetation across Round Island because of limited logging and the introduction of invasive herbivores may have led to reduced populations of ground-nesting ants, if such native species existed. The original habitat of Round Island was primarily thick forest, especially in areas of deep soil accumulation, consisting mostly of latan palms (*Latania loddigesii*), Hurricane palms (*Dictyosperma album* var. *conjugatum*), and bottle palms (*Hyophorbe lagenicaulis*) (Cheke and Hume 2008). If native ants did inhabit Round Island, they would have been adapted to moist thick soils beneath a more or less closed canopy, though some of the island may have been more open, such as the summit, in the hypothesised “tortoise lawn” habitats, and in areas with shallower soil (Cheke and Hume 2008). These habitats became exceptionally rare after the loss of the trees and subsequent erosion of soil until habitat regeneration began in the 1980s. The arrival of non-native ants on Round Island may have led to the loss of native species if any remained by that time. Some non-native ants present in the main island of Mauritius may have faced delays in subsequently colonising Round Island due to its relative inaccessibility. There are at least 53 recorded species of ants found on mainland Mauritius that have not colonised Round Island, most of which are non-native (unpubl. data).

Table S1. Ant species recorded on Round Island with life history information and their use in this study; for the ‘diet analysis’ column, numbers in parentheses indicate the number of samples with diet data after data cleaning.

| Species | Subfamily | Native range | Assumed diet | Colony structure | First record on Round Island | Diet analysis |
| --- | --- | --- | --- | --- | --- | --- |
| *Brachymyrmex cordemoyi* | Formicinae | Neotropics | Generalist omnivore | Unknown | 2005 | Yes (116) |
| *Camponotus maculatus* | Formicinae | Afrotropics + possibly Mascarenes | Generalist omnivore | Monogyny + monodomy | 2001 | No |
| *Cardiocondyla emeryi* | Myrmicinae | Africa | Generalist omnivore | Polygyny + monodomy | 2005 | Yes (41) |
| *Hypoponera mu03* | Ponerinae | Unknown | Predator | Unknown | 2005 | Yes (3) |
| *Monomorium floricola* | Myrmicinae | SE Asia | Generalist omnivore | Polygyny + polydomy | 2005 | Yes (16) |
| *Nylanderia bourbonica* | Formicinae | SE Asia | Generalist omnivore | Polygyny + polydomy | 1975 | Yes (35) |
| *Pheidole indica* | Myrmicinae | Unknown (possibly SE Asia) | Generalist omnivore | Polygyny + polydomy | 2004 | Yes (18) |
| *Pheidole megacephala* | Myrmicinae | Afrotropics | Generalist omnivore | Polygyny + unicolonial | 2005 | Yes (201) |
| *Pheidole parva* | Myrmicinae | SE Asia | Generalist omnivore | Unknown | 2019 (this study) | Yes (11) |
| *Strumigenys simoni* | Myrmicinae | Afrotropics | Specialist predator | Unknown | Unknown | Yes (40) |
| *Syllophopsis sechellensis* | Myrmicinae | Unknown | Unknown | Unknown | 2019 (this study) | No |
| *Tapinoma melanocephalum* | Dolichoderinae | Unknown | Generalist omnivore | Polygyny + unicolonial | Unknown | No |
| *Tapinoma subtile* | Dolichoderinae | Afrotropics + possibly Mascarenes | Unknown | Unknown | Unknown | Yes (43) |
| *Technomyrmex albipes* | Dolichoderinae | Possibly SE Asia | Generalist omnivore | Polygyny | 2005 | No |
| *Technomyrmex pallipes* | Dolichoderinae | Afrotropics | Generalist omnivore | Unknown | 1978 | No |
| *Technomyrmex vitiensis* | Dolichoderinae | Unknown | Generalist omnivore | Unknown | 2005 | Yes (10) |
| *Tetramorium bicarinatum* | Myrmicinae | SE Asia | Generalist omnivore | Polygyny + unicolonial | 2019 (this study) | No |
| *Tetramorium simillimum* | Myrmicinae | Afrotropics | Generalist omnivore | Polygyny | 1975 | Yes (33) |

Invertebrates on Round Island were sampled in two ways: pitfall trapping and hand collection. All ants for dietary metabarcoding were captured by hand. Pitfall trapping was used to measure the invertebrate community diversity on Round Island in each randomly generated 2 x 2 m quadrat. A large number of quadrats were generated on areas of bare rock, and this is an important habitat of Round Island. We therefore used canvas-skirted traps fixed into the rock with masonry nails to sample these areas (Figure S1). We found that different trap types (canvas vs. ground) approached a significant difference in the average number of invertebrates captured (t-test: *t* = -1.94, df = 74, p = 0.056), though the effect size of this result was very small (Hedges’ *g* = -0.012). The composition significantly differed between trap types (MGLM in mvabund: LRT = 44.84, p = 0.006), but our preliminary analysis revealed that these differences were driven by *P. megacephala* abundance between trap types (Wilcoxon rank sum: *W* = 427.5, p = 0.002, median difference between all pairs = -0.4), which is probably best explained by broad habitat preferences of *P. megacephala* than the effectiveness of the traps themselves. Once *P. megacephala* was removed from the analysis, trap type did not significantly affect the number (*W* = 627, p = 0.346, median difference between pairs = -0.25) or composition (MGLM: Dev = 30.01, p = 0.107) of invertebrates captured and we therefore treated different trap types identically. For a summary of replication, see Table S2.


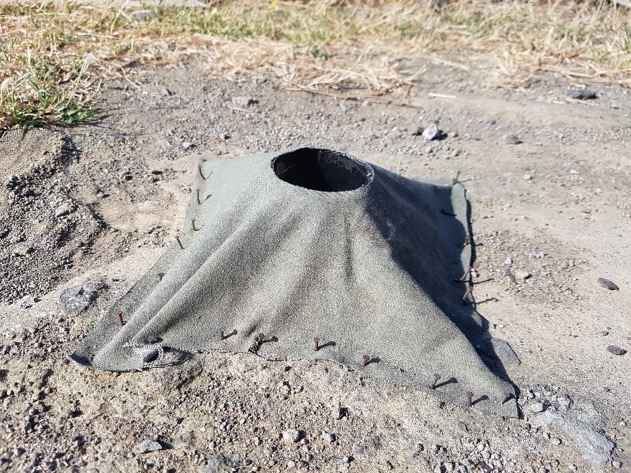


Figure S1. Skirted canvas pitfall trap suitable for rocky substrates.

Samples consisted primarily of non-native ants in most quadrats, with *Pheidole megacephala* numerically dominating the community (Figure S2).

Figure S2. Abundance of invertebrates captured in 77 pitfall traps over Round Island. Ants are given lower taxonomic information, whilst other taxa were identified to order or class. Note the logarithmic scale.

Table S2. The ecological principles of interest and scales at which they are examined in the present study, including replicates for each factor.

| **Factor of interest** | **Scale of inference** | **Scale at which the factor of interest is applied** | **Number of replicates at the appropriate scale** |
| --- | --- | --- | --- |
| Predatory intraguild interactions | Ecological community (of ants) | Ecological community (of ants on Round Island) | 755 ants with dietary data (from 12 species) |
| Shared food resources | Ecological community (of ants) | Ecological community (of ants on Round Island) | 755 ants with dietary data (from 12 species) |
| Foraging behaviour at baits | Species | Species | 21 food baits |
| Prey preferences | Species | Species | 755 ants with dietary data paired with 77 pitfall traps |
| Spatial co-occurrence of species | Quadrat | Quadrat | 77 quadrats (hand searching for ant species) |

Appendix S2. Detailed molecular pipeline and PCR primers.

Polymerase chain reactions (PCR) amplified dietary DNA using invertebrate primers BerenF-LuthienR (Cuff et al. 2021) and AntExF-AntExR (Table S3, Figure S3), which amplify 314bp and 214bp fragments of the mitochondrial COI gene, respectively, and plant primers UniPlant (Moorhouse-Gann et al. 2018), which amplify plant DNA. The use of three primer pairs mitigated the problems associated with using a single primer pair (Tercel et al. 2021, Cuff et al. 2023). Primers were uniquely labelled using 8bp molecular identification tags (MID-tags) to identify samples bioinformatically. PCR product sizes and concentrations were determined via QIAxcel and subsequently pooled for equimolarity and cleaned: each pool was cleaned using SPRIselect beads (Beckman Coulter, Brea, USA), with a left-side size selection using a 1:1 ratio. Libraries were prepared for Illumina sequencing using NEXTflex™ Rapid DNA-Seq Kit following the manufacturer’s instructions (Bioo Scientific Corp, Austin, TX, United States). To confirm fragment size and correct ligation of adapters, libraries were run on an Agilent 4200 TapeStation with D1000 ScreenTape (Agilent Technologies, Waldbronn). PCR products from each primer pair were sequenced separately using an Illumina MiSeq. BerenF-LuthienR amplicons were sequenced on a V3 cartridge using 2 x 300 bp reads, and both AntExF-AntExR and UniPlant were sequenced on separate V2 cartridges using 2 x 250 bp reads. We took forward 1,241 samples (including positives and negatives) with the invertebrate primers and 811 samples with UniPlant. The Illumina sequencing runs generated an average read depth of 8,151 for AntEx, 12,993 for Beren-Lutien, and 19,381 for UniPlant primers, respectively. Bioinformatics and data cleaning followed Tercel (2023): FastP (Chen et al. 2018) was used to check the quality of reads, discard poor quality reads (< Q30, < 125bp long or too many unqualified bases, denoted by “N”), trim reads to a minimum length specific to each primer pair and merge read pairs from MiSeq files (R1 and R2). Read pairs were assigned to samples and demultiplexed using Mothur v1.39.5 (Schloss et al. 2009), after which MID-tag and primer ends were removed.

We used two universal animal primers to reveal the carnivorous aspect of ant diet (Table S3). The first was Beren-Luthien, a universal animal primer designed to amplify a very broad range of invertebrates (Cuff et al. 2021). We aimed for the second primer pair to exclude ant DNA, thus overcoming the problems associated with excessive host reads flooding the dietary data (Cuff et al. 2022). No such primer pair existed, so we designed a new primer pair within the COI barcoding region (Folmer et al. 1994) to amplify as many invertebrates and vertebrates as possible whilst excluding ant DNA. Termed AntEx, our *in silico* tests using PrimerMiner (Elbrecht and Leese 2017) showed a complete exclusion of ant DNA whilst still amplifying a broad range of different invertebrates and vertebrates (Table S1, Figure S3). We thus took this forward for *in vitro* testing and ultimately sequencing.

Table S3. Primers used in the current study. AntEx primers were designed for the current study, targeting a 214bp amplicon of the mitochondrial COI gene. BerenF-LuthienR and UniPlant primers amplify 314bp and 250bp amplicons from the COI and ITS2 markers, respectively.

| Primer | Sequence (5’-3’) | Source | Direction | Base pairs |
| --- | --- | --- | --- | --- |
| AntExF  (ant exclusion) | TAATTGGDGGHTTYGGWAAYTG | This study | Forward | 21 |
| AntExR  (ant exclusion) | CCTAAAATTGADGADAYHCCWGC | This study | Reverse | 22 |
|  |  |  |  |  |
| BerenF  (general animal) | CAGGWTGAACWGTWTAYCCYCC | (Cuff *et al.*, 2021) | Forward | 22 |
| LuthienR  (general animal) | ACTTCWGGRTGWCCAAARAAYCA | (Folmer *et al.*, 1994) | Reverse | 23 |
|  |  |  |  |  |
| UniPlantF  (general plant) | TGTGAATTGCARRATYCMG | (Moorhouse-Gann *et al.*, 2018) | Forward | 19 |
| UniPlantR  (general plant) | CCCGHYTGAYYTGRGGTCDC | (Moorhouse-Gann *et al.*, 2018) | Reverse | 20 |

Figure S3. Results of *in silico* amplification testing using PrimerMiner to determine the amplification efficiency of AntEx for a range of different taxa present on Round Island.

Appendix S3. Photo attributions for Figure 2

Photos for Figure 2 in the main document were downloaded from AntWeb.org v8.106.1 under CC-BY 4.0. Photo attribution (“specimen code, photographer”): *B. cordemoyi* (CASENT0103230, April Noble); *C. emeryi* (ANTWEB1048512, Bernhard Seifert); *H. mu03* (CASENT0060381, April Noble); *M. floricola* (CASENT0060702, April Noble); *N. bourbonica* (CASENT0060259, April Noble); *P. indica* (CASENT0264427, Estella Ortega); *P. megacephala* (CASENT0104990, Michele Esposito); *P. parva* (CASENT0055997, April Noble); *S. simoni* (CASENT0005997, April Noble); *T. subtile* (CASENT0132840, Erin Prado); *T. vitiensis* (CASENT0103244, April Noble); *T. simillimum* (CASENT0102390, April Noble).

Appendix S4. Diet composition and NMDS plots.

Our MGLMMs showed that diet composition varied significantly between season and ant species, and we therefore conducted separate compositional analyses of the diet of the ant community for both wet and dry seasons. Ant-ant dietary composition varied significantly between consumer species in both the wet and dry seasons (wet: LRT = 440.9, p = <0.001; dry: LRT = 279.4, p = 0.001). Dietary variation was visualised using non-metric multidimensional scaling analysis (NMDS) using the “metaMDS” function in the “vegan” R package (Oksanen et al. 2019) with Jaccard distance and was plotted using “ggplot2” (Wickham 2016) (Figure S4).


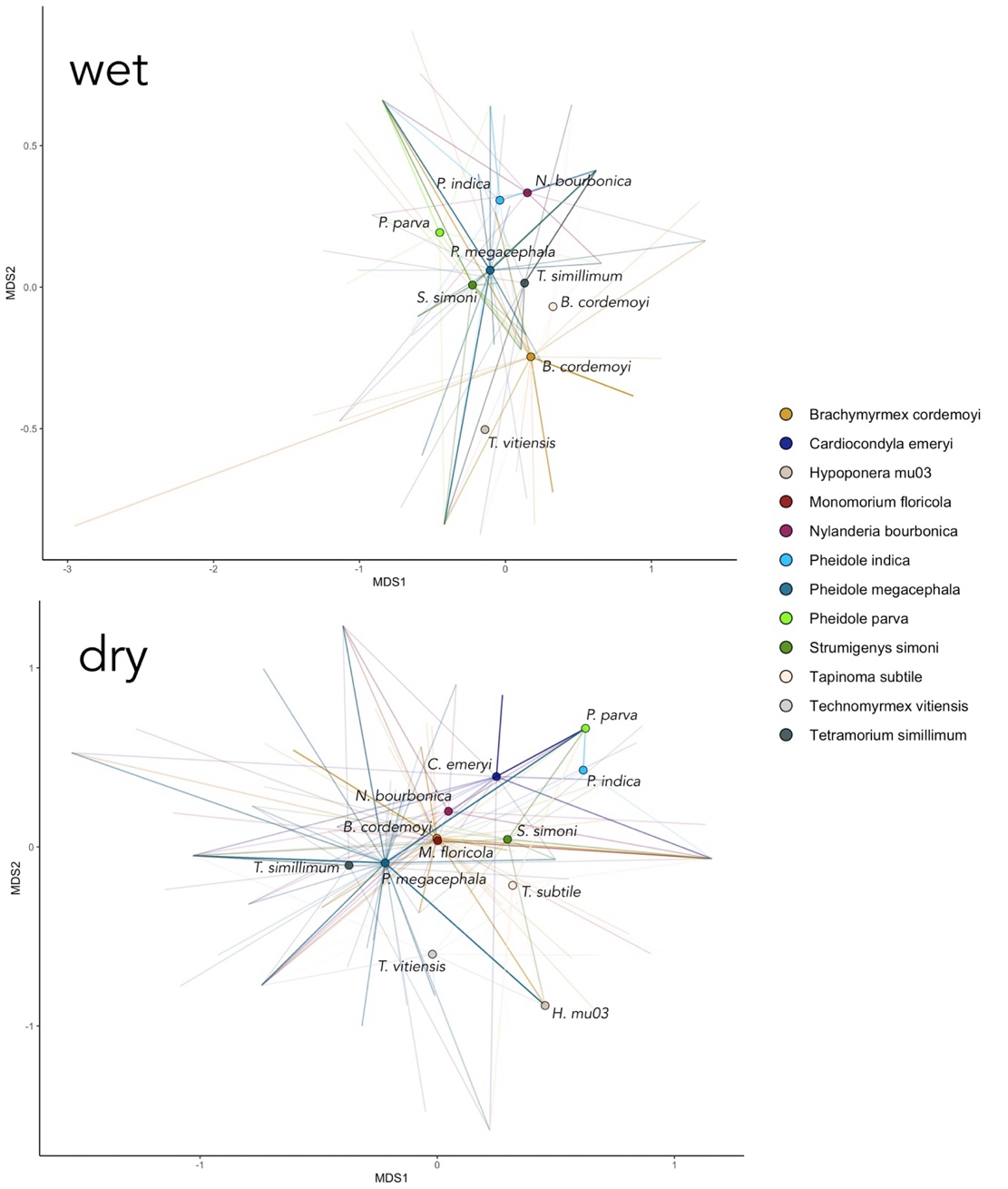


Figure S4. Ant species diet composition of other ants consumed between wet (**a)** and dry (**b**) seasons visualised using non-metric multidimensional scaling (NMDS). Colours denote different species of ant consumers, and the large points show the associated mean dietary composition of a given species and are annotated with binomial species names beside each; terminal ends of lines represent the dietary composition of individual ants and are connected to their associated centroid.

Appendix S5. Competitive interactions.


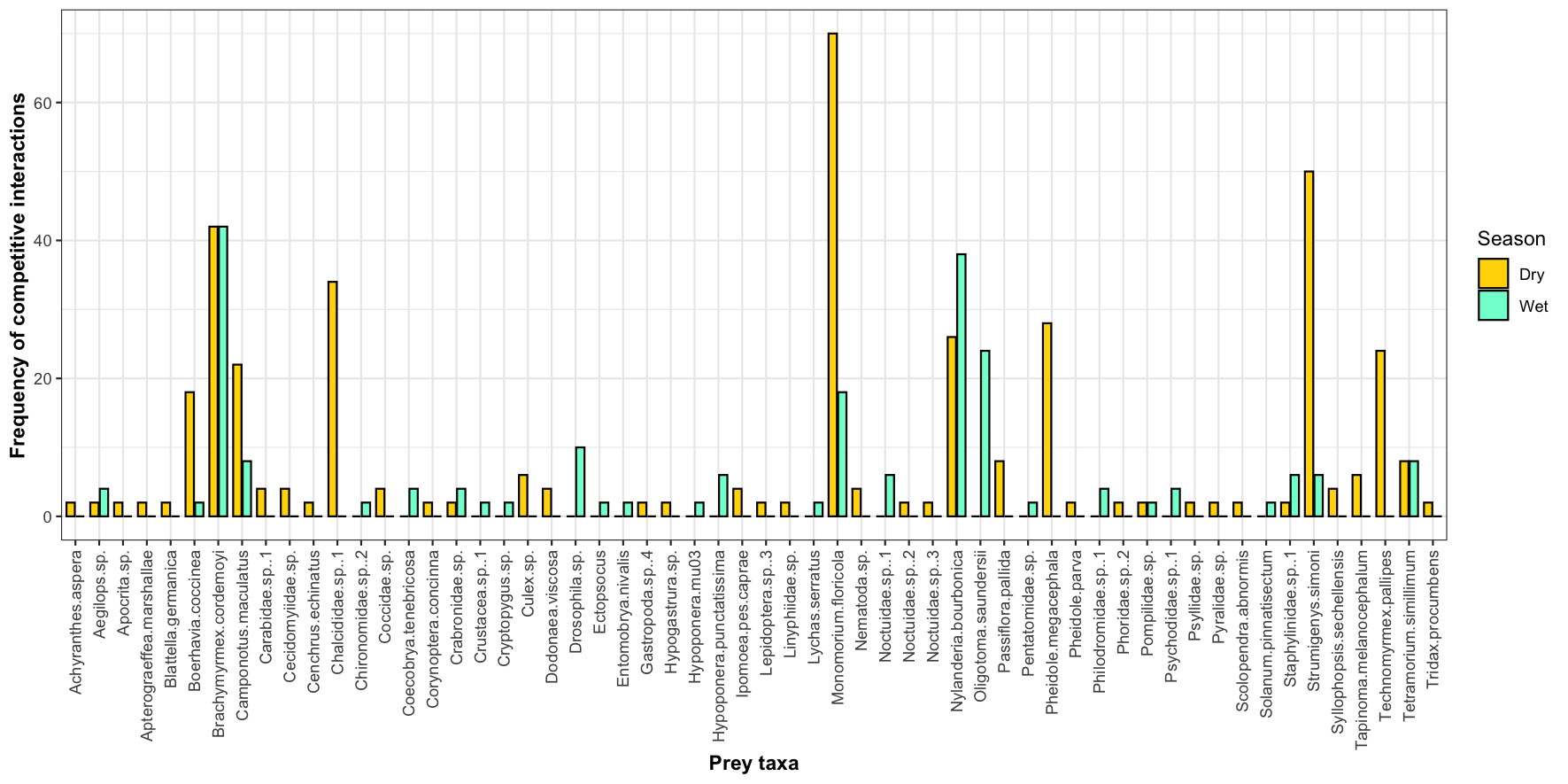


Figure S5: The frequency with which ants were competing for different prey. Frequencies denote the number of occurrences of each prey taxon in competitive interactions between ants, with yellow representing the dry season and green the wet season.


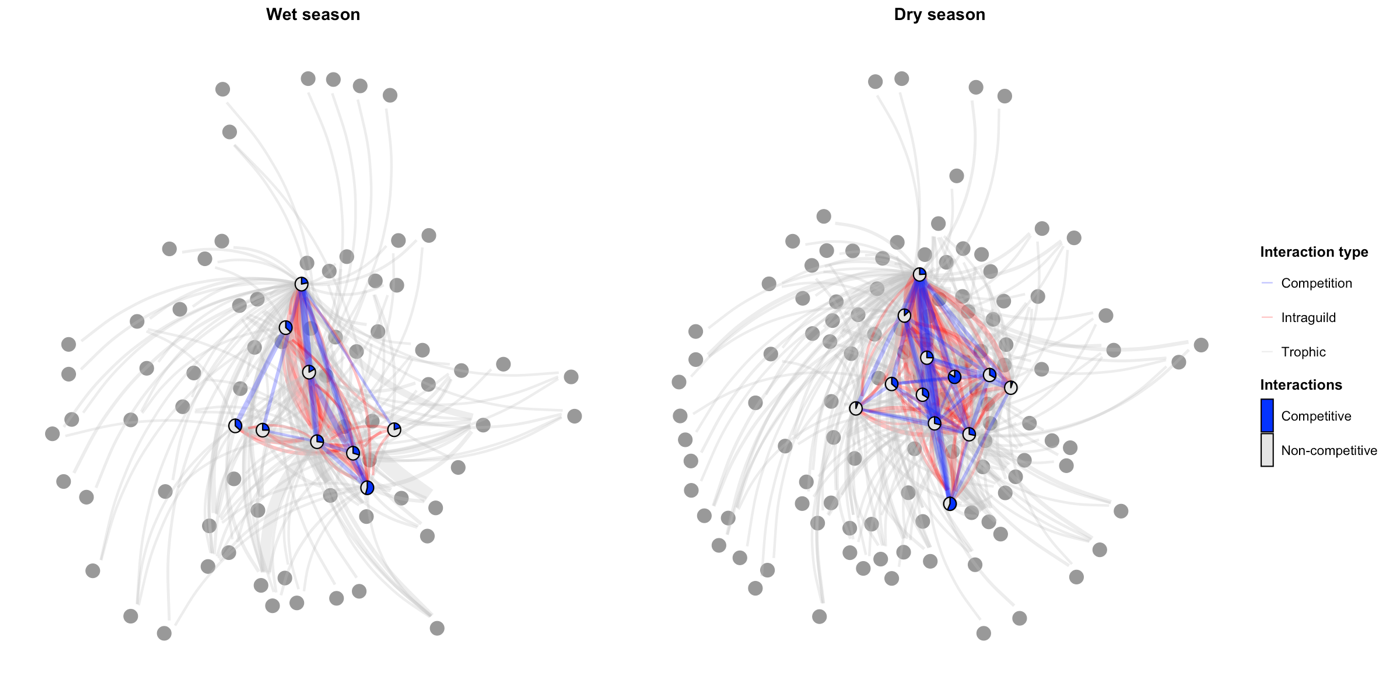


Figure S6: Networks of direct ant-non-ant-prey (trophic; curved grey links) and ant-ant (intraguild; curved red links), and indirect second order ant-ant (competition; straight blue links) interactions. Pie charts represent ant nodes, with the ratio of grey to blue denoting the proportion of non-competitive (i.e., prey not shared with another ant in the same space and time) to competitive (i.e., prey shared with another ant in the same space and time) interactions with prey, respectively. The plots below show the frequency of competitive interactions between different ant species. Plots to the left are based on wet season data, and the right represent the dry season.

Table S4: Comparison of the competitive richness of each ant taxon across the study. Values are the estimate, standard error, z-value and p-value of a GLMM.

|  | *Cardiocondyla emeryi* | *Hypoponera mu03* | *Monomorium floricola* | *Nylanderia bourbonica* | *Pheidole indica* | *Pheidole megacephala* | *Pheidole parva* | *Strumigenys simoni* | *Tapinoma subtile* | *Technomyrmex vitiensis* | *Tetramorium simillimum* |
| --- | --- | --- | --- | --- | --- | --- | --- | --- | --- | --- | --- |
| *Brachymyrmex cordemoyi* | estimate = -0.416 ± 0.299, z ratio = -1.391, p = 0.965 | estimate = 0.842 ± 0.596, z ratio = 1.412, p = 0.961 | estimate = -0.055 ± 0.242, z ratio = -0.227, p = 1 | estimate = 0.092 ± 0.179, z ratio = 0.512, p = 1 | estimate = 0.103 ± 0.245, z ratio = 0.421, p = 1 | estimate = -0.116 ± 0.121, z ratio = -0.965, p = 0.998 | estimate = -0.08 ± 0.332, z ratio = -0.24, p = 1 | estimate = -0.325 ± 0.185, z ratio = -1.755, p = 0.842 | estimate = -0.419 ± 0.197, z ratio = -2.125, p = 0.605 | estimate = 0.15 ± 0.392, z ratio = 0.384, p = 1 | estimate = 0.095 ± 0.168, z ratio = 0.562, p = 1 |
| *Cardiocondyla emeryi* | NA | estimate = 1.257 ± 0.653, z ratio = 1.925, p = 0.743 | estimate = 0.361 ± 0.338, z ratio = 1.069, p = 0.996 | estimate = 0.507 ± 0.323, z ratio = 1.573, p = 0.919 | estimate = 0.519 ± 0.362, z ratio = 1.434, p = 0.957 | estimate = 0.299 ± 0.288, z ratio = 1.04, p = 0.997 | estimate = 0.336 ± 0.425, z ratio = 0.791, p = 1 | estimate = 0.09 ± 0.317, z ratio = 0.285, p = 1 | estimate = -0.003 ± 0.308, z ratio = -0.011, p = 1 | estimate = 0.566 ± 0.473, z ratio = 1.198, p = 0.989 | estimate = 0.51 ± 0.306, z ratio = 1.666, p = 0.884 |
| *Hypoponera mu03* | NA | NA | estimate = -0.897 ± 0.629, z ratio = -1.426, p = 0.959 | estimate = -0.75 ± 0.606, z ratio = -1.239, p = 0.986 | estimate = -0.739 ± 0.625, z ratio = -1.182, p = 0.99 | estimate = -0.958 ± 0.593, z ratio = -1.615, p = 0.904 | estimate = -0.921 ± 0.663, z ratio = -1.389, p = 0.966 | estimate = -1.167 ± 0.614, z ratio = -1.902, p = 0.758 | estimate = -1.261 ± 0.616, z ratio = -2.048, p = 0.66 | estimate = -0.691 ± 0.699, z ratio = -0.989, p = 0.998 | estimate = -0.747 ± 0.606, z ratio = -1.233, p = 0.986 |
| *Monomorium floricola* | NA | NA | NA | estimate = 0.146 ± 0.265, z ratio = 0.552, p = 1 | estimate = 0.158 ± 0.311, z ratio = 0.508, p = 1 | estimate = -0.062 ± 0.232, z ratio = -0.265, p = 1 | estimate = -0.025 ± 0.389, z ratio = -0.064, p = 1 | estimate = -0.271 ± 0.263, z ratio = -1.027, p = 0.997 | estimate = -0.364 ± 0.272, z ratio = -1.339, p = 0.974 | estimate = 0.205 ± 0.438, z ratio = 0.469, p = 1 | estimate = 0.149 ± 0.262, z ratio = 0.57, p = 1 |
| *Nylanderia bourbonica* | NA | NA | NA | NA | estimate = 0.011 ± 0.267, z ratio = 0.043, p = 1 | estimate = -0.208 ± 0.172, z ratio = -1.21, p = 0.988 | estimate = -0.171 ± 0.351, z ratio = -0.487, p = 1 | estimate = -0.417 ± 0.222, z ratio = -1.88, p = 0.772 | estimate = -0.511 ± 0.235, z ratio = -2.172, p = 0.57 | estimate = 0.059 ± 0.405, z ratio = 0.145, p = 1 | estimate = 0.003 ± 0.212, z ratio = 0.014, p = 1 |
| *Pheidole indica* | NA | NA | NA | NA | NA | estimate = -0.219 ± 0.237, z ratio = -0.924, p = 0.999 | estimate = -0.183 ± 0.386, z ratio = -0.473, p = 1 | estimate = -0.428 ± 0.285, z ratio = -1.503, p = 0.94 | estimate = -0.522 ± 0.289, z ratio = -1.805, p = 0.816 | estimate = 0.047 ± 0.419, z ratio = 0.113, p = 1 | estimate = -0.008 ± 0.271, z ratio = -0.031, p = 1 |
| *Pheidole megacephala* | NA | NA | NA | NA | NA | NA | estimate = 0.037 ± 0.329, z ratio = 0.112, p = 1 | estimate = -0.209 ± 0.179, z ratio = -1.166, p = 0.991 | estimate = -0.303 ± 0.187, z ratio = -1.619, p = 0.902 | estimate = 0.267 ± 0.387, z ratio = 0.69, p = 1 | estimate = 0.211 ± 0.161, z ratio = 1.312, p = 0.978 |
| *Pheidole parva* | NA | NA | NA | NA | NA | NA | NA | estimate = -0.246 ± 0.361, z ratio = -0.681, p = 1 | estimate = -0.34 ± 0.356, z ratio = -0.954, p = 0.999 | estimate = 0.23 ± 0.497, z ratio = 0.462, p = 1 | estimate = 0.174 ± 0.35, z ratio = 0.498, p = 1 |
| *Strumigenys simoni* | NA | NA | NA | NA | NA | NA | NA | NA | estimate = -0.094 ± 0.235, z ratio = -0.398, p = 1 | estimate = 0.476 ± 0.419, z ratio = 1.137, p = 0.993 | estimate = 0.42 ± 0.211, z ratio = 1.991, p = 0.7 |
| *Tapinoma subtile* | NA | NA | NA | NA | NA | NA | NA | NA | NA | estimate = 0.57 ± 0.42, z ratio = 1.356, p = 0.971 | estimate = 0.514 ± 0.219, z ratio = 2.343, p = 0.446 |
| *Technomyrmex vitiensis* | NA | NA | NA | NA | NA | NA | NA | NA | NA | NA | estimate = -0.056 ± 0.411, z ratio = -0.136, p = 1 |

Table S5: Comparison of the frequency of competitive interactions that each ant taxon engaged in across the study. Values are the estimate, standard error, z-value and p-value of a GLMM.

|  | *Cardiocondyla emeryi* | *Hypoponera mu03* | *Monomorium floricola* | *Nylanderia bourbonica* | *Pheidole indica* | *Pheidole megacephala* | *Pheidole parva* | *Strumigenys simoni* | *Tapinoma subtile* | *Technomyrmex vitiensis* | *Tetramorium simillimum* |
| --- | --- | --- | --- | --- | --- | --- | --- | --- | --- | --- | --- |
| *Brachymyrmex cordemoyi* | estimate = -0.091 ± 0.251, z ratio = -0.362, p = 1 | estimate = 0.925 ± 0.598, z ratio = 1.547, p = 0.927 | estimate = 0.121 ± 0.223, z ratio = 0.543, p = 1 | estimate = 0.145 ± 0.165, z ratio = 0.879, p = 0.999 | estimate = 0.068 ± 0.227, z ratio = 0.3, p = 1 | estimate = -0.175 ± 0.106, z ratio = -1.651, p = 0.89 | estimate = -0.131 ± 0.291, z ratio = -0.449, p = 1 | estimate = -0.484 ± 0.155, z ratio = -3.125, p = 0.077 | estimate = -0.39 ± 0.167, z ratio = -2.334, p = 0.452 | estimate = -0.261 ± 0.312, z ratio = -0.836, p = 1 | estimate = 0.148 ± 0.147, z ratio = 1.004, p = 0.998 |
| *Cardiocondyla emeryi* | NA | estimate = 1.016 ± 0.638, z ratio = 1.592, p = 0.913 | estimate = 0.212 ± 0.29, z ratio = 0.731, p = 1 | estimate = 0.236 ± 0.277, z ratio = 0.851, p = 0.999 | estimate = 0.159 ± 0.318, z ratio = 0.501, p = 1 | estimate = -0.084 ± 0.239, z ratio = -0.351, p = 1 | estimate = -0.04 ± 0.358, z ratio = -0.111, p = 1 | estimate = -0.393 ± 0.259, z ratio = -1.518, p = 0.936 | estimate = -0.299 ± 0.251, z ratio = -1.193, p = 0.99 | estimate = -0.17 ± 0.382, z ratio = -0.445, p = 1 | estimate = 0.239 ± 0.254, z ratio = 0.939, p = 0.999 |
| *Hypoponera mu03* | NA | NA | estimate = -0.804 ± 0.627, z ratio = -1.283, p = 0.981 | estimate = -0.78 ± 0.607, z ratio = -1.286, p = 0.981 | estimate = -0.857 ± 0.624, z ratio = -1.375, p = 0.968 | estimate = -1.1 ± 0.595, z ratio = -1.848, p = 0.791 | estimate = -1.056 ± 0.651, z ratio = -1.622, p = 0.901 | estimate = -1.409 ± 0.609, z ratio = -2.313, p = 0.467 | estimate = -1.315 ± 0.612, z ratio = -2.15, p = 0.586 | estimate = -1.186 ± 0.663, z ratio = -1.789, p = 0.824 | estimate = -0.778 ± 0.606, z ratio = -1.284, p = 0.981 |
| *Monomorium floricola* | NA | NA | NA | estimate = 0.024 ± 0.248, z ratio = 0.097, p = 1 | estimate = -0.053 ± 0.29, z ratio = -0.181, p = 1 | estimate = -0.296 ± 0.212, z ratio = -1.397, p = 0.964 | estimate = -0.251 ± 0.344, z ratio = -0.732, p = 1 | estimate = -0.604 ± 0.233, z ratio = -2.599, p = 0.28 | estimate = -0.511 ± 0.237, z ratio = -2.157, p = 0.581 | estimate = -0.382 ± 0.363, z ratio = -1.052, p = 0.996 | estimate = 0.027 ± 0.235, z ratio = 0.115, p = 1 |
| *Nylanderia bourbonica* | NA | NA | NA | NA | estimate = -0.077 ± 0.25, z ratio = -0.307, p = 1 | estimate = -0.32 ± 0.157, z ratio = -2.029, p = 0.673 | estimate = -0.276 ± 0.312, z ratio = -0.884, p = 0.999 | estimate = -0.628 ± 0.193, z ratio = -3.252, p = 0.052 | estimate = -0.535 ± 0.205, z ratio = -2.609, p = 0.274 | estimate = -0.406 ± 0.329, z ratio = -1.235, p = 0.986 | estimate = 0.003 ± 0.191, z ratio = 0.015, p = 1 |
| *Pheidole indica* | NA | NA | NA | NA | NA | estimate = -0.243 ± 0.22, z ratio = -1.104, p = 0.995 | estimate = -0.199 ± 0.347, z ratio = -0.574, p = 1 | estimate = -0.552 ± 0.255, z ratio = -2.164, p = 0.576 | estimate = -0.458 ± 0.259, z ratio = -1.766, p = 0.837 | estimate = -0.329 ± 0.339, z ratio = -0.97, p = 0.998 | estimate = 0.08 ± 0.249, z ratio = 0.32, p = 1 |
| *Pheidole megacephala* | NA | NA | NA | NA | NA | NA | estimate = 0.044 ± 0.286, z ratio = 0.154, p = 1 | estimate = -0.309 ± 0.146, z ratio = -2.12, p = 0.608 | estimate = -0.215 ± 0.153, z ratio = -1.402, p = 0.963 | estimate = -0.086 ± 0.307, z ratio = -0.281, p = 1 | estimate = 0.322 ± 0.137, z ratio = 2.348, p = 0.442 |
| *Pheidole parva* | NA | NA | NA | NA | NA | NA | NA | estimate = -0.353 ± 0.31, z ratio = -1.137, p = 0.993 | estimate = -0.259 ± 0.292, z ratio = -0.889, p = 0.999 | estimate = -0.13 ± 0.411, z ratio = -0.317, p = 1 | estimate = 0.278 ± 0.304, z ratio = 0.915, p = 0.999 |
| *Strumigenys simoni* | NA | NA | NA | NA | NA | NA | NA | NA | estimate = 0.094 ± 0.19, z ratio = 0.493, p = 1 | estimate = 0.223 ± 0.334, z ratio = 0.667, p = 1 | estimate = 0.631 ± 0.172, z ratio = 3.677, p = 0.013 |
| *Tapinoma subtile* | NA | NA | NA | NA | NA | NA | NA | NA | NA | estimate = 0.129 ± 0.335, z ratio = 0.385, p = 1 | estimate = 0.538 ± 0.182, z ratio = 2.956, p = 0.122 |
| *Technomyrmex vitiensis* | NA | NA | NA | NA | NA | NA | NA | NA | NA | NA | estimate = 0.409 ± 0.33, z ratio = 1.237, p = 0.986 |

Table S6: Comparison of the percentage the richness of ant-prey interactions that were shared with other ants in the same space and time between each ant taxon across the study. Values are the estimate, standard error, t-value and p-value of a LMM.

|  | *Cardiocondyla emeryi* | *Hypoponera mu03* | *Monomorium floricola* | *Nylanderia bourbonica* | *Pheidole indica* | *Pheidole megacephala* | *Pheidole parva* | *Strumigenys simoni* | *Tapinoma subtile* | *Technomyrmex vitiensis* | *Tetramorium simillimum* |
| --- | --- | --- | --- | --- | --- | --- | --- | --- | --- | --- | --- |
| *Brachymyrmex cordemoyi* | estimate = -0.416 ± 0.299, z ratio = -1.391, p = 0.965 | estimate = 0.842 ± 0.596, z ratio = 1.412, p = 0.961 | estimate = -0.055 ± 0.242, z ratio = -0.227, p = 1 | estimate = 0.092 ± 0.179, z ratio = 0.512, p = 1 | estimate = 0.103 ± 0.245, z ratio = 0.421, p = 1 | estimate = -0.116 ± 0.121, z ratio = -0.965, p = 0.998 | estimate = -0.08 ± 0.332, z ratio = -0.24, p = 1 | estimate = -0.325 ± 0.185, z ratio = -1.755, p = 0.842 | estimate = -0.419 ± 0.197, z ratio = -2.125, p = 0.605 | estimate = 0.15 ± 0.392, z ratio = 0.384, p = 1 | estimate = 0.095 ± 0.168, z ratio = 0.562, p = 1 |
| *Cardiocondyla emeryi* | NA | estimate = 1.257 ± 0.653, z ratio = 1.925, p = 0.743 | estimate = 0.361 ± 0.338, z ratio = 1.069, p = 0.996 | estimate = 0.507 ± 0.323, z ratio = 1.573, p = 0.919 | estimate = 0.519 ± 0.362, z ratio = 1.434, p = 0.957 | estimate = 0.299 ± 0.288, z ratio = 1.04, p = 0.997 | estimate = 0.336 ± 0.425, z ratio = 0.791, p = 1 | estimate = 0.09 ± 0.317, z ratio = 0.285, p = 1 | estimate = -0.003 ± 0.308, z ratio = -0.011, p = 1 | estimate = 0.566 ± 0.473, z ratio = 1.198, p = 0.989 | estimate = 0.51 ± 0.306, z ratio = 1.666, p = 0.884 |
| *Hypoponera mu03* | NA | NA | estimate = -0.897 ± 0.629, z ratio = -1.426, p = 0.959 | estimate = -0.75 ± 0.606, z ratio = -1.239, p = 0.986 | estimate = -0.739 ± 0.625, z ratio = -1.182, p = 0.99 | estimate = -0.958 ± 0.593, z ratio = -1.615, p = 0.904 | estimate = -0.921 ± 0.663, z ratio = -1.389, p = 0.966 | estimate = -1.167 ± 0.614, z ratio = -1.902, p = 0.758 | estimate = -1.261 ± 0.616, z ratio = -2.048, p = 0.66 | estimate = -0.691 ± 0.699, z ratio = -0.989, p = 0.998 | estimate = -0.747 ± 0.606, z ratio = -1.233, p = 0.986 |
| *Monomorium floricola* | NA | NA | NA | estimate = 0.146 ± 0.265, z ratio = 0.552, p = 1 | estimate = 0.158 ± 0.311, z ratio = 0.508, p = 1 | estimate = -0.062 ± 0.232, z ratio = -0.265, p = 1 | estimate = -0.025 ± 0.389, z ratio = -0.064, p = 1 | estimate = -0.271 ± 0.263, z ratio = -1.027, p = 0.997 | estimate = -0.364 ± 0.272, z ratio = -1.339, p = 0.974 | estimate = 0.205 ± 0.438, z ratio = 0.469, p = 1 | estimate = 0.149 ± 0.262, z ratio = 0.57, p = 1 |
| *Nylanderia bourbonica* | NA | NA | NA | NA | estimate = 0.011 ± 0.267, z ratio = 0.043, p = 1 | estimate = -0.208 ± 0.172, z ratio = -1.21, p = 0.988 | estimate = -0.171 ± 0.351, z ratio = -0.487, p = 1 | estimate = -0.417 ± 0.222, z ratio = -1.88, p = 0.772 | estimate = -0.511 ± 0.235, z ratio = -2.172, p = 0.57 | estimate = 0.059 ± 0.405, z ratio = 0.145, p = 1 | estimate = 0.003 ± 0.212, z ratio = 0.014, p = 1 |
| *Pheidole indica* | NA | NA | NA | NA | NA | estimate = -0.219 ± 0.237, z ratio = -0.924, p = 0.999 | estimate = -0.183 ± 0.386, z ratio = -0.473, p = 1 | estimate = -0.428 ± 0.285, z ratio = -1.503, p = 0.94 | estimate = -0.522 ± 0.289, z ratio = -1.805, p = 0.816 | estimate = 0.047 ± 0.419, z ratio = 0.113, p = 1 | estimate = -0.008 ± 0.271, z ratio = -0.031, p = 1 |
| *Pheidole megacephala* | NA | NA | NA | NA | NA | NA | estimate = 0.037 ± 0.329, z ratio = 0.112, p = 1 | estimate = -0.209 ± 0.179, z ratio = -1.166, p = 0.991 | estimate = -0.303 ± 0.187, z ratio = -1.619, p = 0.902 | estimate = 0.267 ± 0.387, z ratio = 0.69, p = 1 | estimate = 0.211 ± 0.161, z ratio = 1.312, p = 0.978 |
| *Pheidole parva* | NA | NA | NA | NA | NA | NA | NA | estimate = -0.246 ± 0.361, z ratio = -0.681, p = 1 | estimate = -0.34 ± 0.356, z ratio = -0.954, p = 0.999 | estimate = 0.23 ± 0.497, z ratio = 0.462, p = 1 | estimate = 0.174 ± 0.35, z ratio = 0.498, p = 1 |
| *Strumigenys simoni* | NA | NA | NA | NA | NA | NA | NA | NA | estimate = -0.094 ± 0.235, z ratio = -0.398, p = 1 | estimate = 0.476 ± 0.419, z ratio = 1.137, p = 0.993 | estimate = 0.42 ± 0.211, z ratio = 1.991, p = 0.7 |
| *Tapinoma subtile* | NA | NA | NA | NA | NA | NA | NA | NA | NA | estimate = 0.57 ± 0.42, z ratio = 1.356, p = 0.971 | estimate = 0.514 ± 0.219, z ratio = 2.343, p = 0.446 |
| *Technomyrmex vitiensis* | NA | NA | NA | NA | NA | NA | NA | NA | NA | NA | estimate = -0.056 ± 0.411, z ratio = -0.136, p = 1 |

Table S7: Comparison of the percentage of the frequency of ant-prey interactions that were shared with other ants in the same space and time between each ant taxon across the study. Values are the estimate, standard error, t-value and p-value of a LMM.

|  | *Cardiocondyla emeryi* | *Hypoponera mu03* | *Monomorium floricola* | *Nylanderia bourbonica* | *Pheidole indica* | *Pheidole megacephala* | *Pheidole parva* | *Strumigenys simoni* | *Tapinoma subtile* | *Technomyrmex vitiensis* | *Tetramorium simillimum* |
| --- | --- | --- | --- | --- | --- | --- | --- | --- | --- | --- | --- |
| *Brachymyrmex cordemoyi* | estimate = -0.091 ± 0.251, z ratio = -0.362, p = 1 | estimate = 0.925 ± 0.598, z ratio = 1.547, p = 0.927 | estimate = 0.121 ± 0.223, z ratio = 0.543, p = 1 | estimate = 0.145 ± 0.165, z ratio = 0.879, p = 0.999 | estimate = 0.068 ± 0.227, z ratio = 0.3, p = 1 | estimate = -0.175 ± 0.106, z ratio = -1.651, p = 0.89 | estimate = -0.131 ± 0.291, z ratio = -0.449, p = 1 | estimate = -0.484 ± 0.155, z ratio = -3.125, p = 0.077 | estimate = -0.39 ± 0.167, z ratio = -2.334, p = 0.452 | estimate = -0.261 ± 0.312, z ratio = -0.836, p = 1 | estimate = 0.148 ± 0.147, z ratio = 1.004, p = 0.998 |
| *Cardiocondyla emeryi* | NA | estimate = 1.016 ± 0.638, z ratio = 1.592, p = 0.913 | estimate = 0.212 ± 0.29, z ratio = 0.731, p = 1 | estimate = 0.236 ± 0.277, z ratio = 0.851, p = 0.999 | estimate = 0.159 ± 0.318, z ratio = 0.501, p = 1 | estimate = -0.084 ± 0.239, z ratio = -0.351, p = 1 | estimate = -0.04 ± 0.358, z ratio = -0.111, p = 1 | estimate = -0.393 ± 0.259, z ratio = -1.518, p = 0.936 | estimate = -0.299 ± 0.251, z ratio = -1.193, p = 0.99 | estimate = -0.17 ± 0.382, z ratio = -0.445, p = 1 | estimate = 0.239 ± 0.254, z ratio = 0.939, p = 0.999 |
| *Hypoponera mu03* | NA | NA | estimate = -0.804 ± 0.627, z ratio = -1.283, p = 0.981 | estimate = -0.78 ± 0.607, z ratio = -1.286, p = 0.981 | estimate = -0.857 ± 0.624, z ratio = -1.375, p = 0.968 | estimate = -1.1 ± 0.595, z ratio = -1.848, p = 0.791 | estimate = -1.056 ± 0.651, z ratio = -1.622, p = 0.901 | estimate = -1.409 ± 0.609, z ratio = -2.313, p = 0.467 | estimate = -1.315 ± 0.612, z ratio = -2.15, p = 0.586 | estimate = -1.186 ± 0.663, z ratio = -1.789, p = 0.824 | estimate = -0.778 ± 0.606, z ratio = -1.284, p = 0.981 |
| *Monomorium floricola* | NA | NA | NA | estimate = 0.024 ± 0.248, z ratio = 0.097, p = 1 | estimate = -0.053 ± 0.29, z ratio = -0.181, p = 1 | estimate = -0.296 ± 0.212, z ratio = -1.397, p = 0.964 | estimate = -0.251 ± 0.344, z ratio = -0.732, p = 1 | estimate = -0.604 ± 0.233, z ratio = -2.599, p = 0.28 | estimate = -0.511 ± 0.237, z ratio = -2.157, p = 0.581 | estimate = -0.382 ± 0.363, z ratio = -1.052, p = 0.996 | estimate = 0.027 ± 0.235, z ratio = 0.115, p = 1 |
| *Nylanderia bourbonica* | NA | NA | NA | NA | estimate = -0.077 ± 0.25, z ratio = -0.307, p = 1 | estimate = -0.32 ± 0.157, z ratio = -2.029, p = 0.673 | estimate = -0.276 ± 0.312, z ratio = -0.884, p = 0.999 | estimate = -0.628 ± 0.193, z ratio = -3.252, p = 0.052 | estimate = -0.535 ± 0.205, z ratio = -2.609, p = 0.274 | estimate = -0.406 ± 0.329, z ratio = -1.235, p = 0.986 | estimate = 0.003 ± 0.191, z ratio = 0.015, p = 1 |
| *Pheidole indica* | NA | NA | NA | NA | NA | estimate = -0.243 ± 0.22, z ratio = -1.104, p = 0.995 | estimate = -0.199 ± 0.347, z ratio = -0.574, p = 1 | estimate = -0.552 ± 0.255, z ratio = -2.164, p = 0.576 | estimate = -0.458 ± 0.259, z ratio = -1.766, p = 0.837 | estimate = -0.329 ± 0.339, z ratio = -0.97, p = 0.998 | estimate = 0.08 ± 0.249, z ratio = 0.32, p = 1 |
| *Pheidole megacephala* | NA | NA | NA | NA | NA | NA | estimate = 0.044 ± 0.286, z ratio = 0.154, p = 1 | estimate = -0.309 ± 0.146, z ratio = -2.12, p = 0.608 | estimate = -0.215 ± 0.153, z ratio = -1.402, p = 0.963 | estimate = -0.086 ± 0.307, z ratio = -0.281, p = 1 | estimate = 0.322 ± 0.137, z ratio = 2.348, p = 0.442 |
| *Pheidole parva* | NA | NA | NA | NA | NA | NA | NA | estimate = -0.353 ± 0.31, z ratio = -1.137, p = 0.993 | estimate = -0.259 ± 0.292, z ratio = -0.889, p = 0.999 | estimate = -0.13 ± 0.411, z ratio = -0.317, p = 1 | estimate = 0.278 ± 0.304, z ratio = 0.915, p = 0.999 |
| *Strumigenys simoni* | NA | NA | NA | NA | NA | NA | NA | NA | estimate = 0.094 ± 0.19, z ratio = 0.493, p = 1 | estimate = 0.223 ± 0.334, z ratio = 0.667, p = 1 | estimate = 0.631 ± 0.172, z ratio = 3.677, p = 0.013 |
| *Tapinoma subtile* | NA | NA | NA | NA | NA | NA | NA | NA | NA | estimate = 0.129 ± 0.335, z ratio = 0.385, p = 1 | estimate = 0.538 ± 0.182, z ratio = 2.956, p = 0.122 |
| *Technomyrmex vitiensis* | NA | NA | NA | NA | NA | NA | NA | NA | NA | NA | estimate = 0.409 ± 0.33, z ratio = 1.237, p = 0.986 |

References

Cheke, A., and J. P. Hume. 2008. Lost Land of the Dodo : An Ecological History of Mauritius, Réunion & Rodrigues. Bloomsbury, London.

Chen, S., Y. Zhou, Y. Chen, and J. Gu. 2018. Fastp: An ultra-fast all-in-one FASTQ preprocessor. Page Bioinformatics.

Cuff, J. P., L. E. Drake, M. P. T. G. Tercel, J. E. Stockdale, P. Orozco‐terWengel, J. R. Bell, I. P. Vaughan, C. T. Müller, and W. O. C. Symondson. 2021. Money spider dietary choice in pre‐ and post‐harvest cereal crops using metabarcoding. Ecological Entomology 46:249–261.

Cuff, J. P., J. J. N. Kitson, D. Hemprich-Bennett, M. P. T. G. Tercel, S. S. Browett, and D. M. Evans. 2023. The predator problem and PCR primers in molecular dietary analysis: Swamped or silenced; depth or breadth? Molecular Ecology Resources 23:41–51.

Deyrup, M. 2017. Ants of Florida: Identification and Natural History. Crc Press, Boca Raton.

Fabricius, J. C. 1793. Entomologia systematica emendata et aucta. Secundum classes, ordines, genera, species, adjectis synonimis, locis observationibus, descriptionibus. C. G. Proft, Hafniae [= Copenhagan].

Moorhouse-Gann, R. J., J. C. Dunn, N. de Vere, M. Goder, N. Cole, H. Hipperson, and W. O. C. Symondson. 2018. New universal ITS2 primers for high-resolution herbivory analyses using DNA metabarcoding in both tropical and temperate zones. Scientific Reports 8:8542.

Schloss, P. D., S. L. Westcott, T. Ryabin, J. R. Hall, M. Hartmann, E. B. Hollister, R. A. Lesniewski, B. B. Oakley, D. H. Parks, C. J. Robinson, J. W. Sahl, B. Stres, G. G. Thallinger, D. J. Van Horn, and C. F. Weber. 2009. Introducing mothur: Open-source, platform-independent, community-supported software for describing and comparing microbial communities. Applied and Environmental Microbiology.

Tercel, M. P. T. G., W. O. C. Symondson, and J. P. Cuff. 2021. The problem of omnivory: A synthesis on omnivory and DNA metabarcoding. Molecular ecology 30:2199–2206.

Wetterer, J. K. 2012. Worldwide spread of the African big-headed ant, Pheidole megacephala (Hymeno-ptera: Formicidae). Myrmecol. News 17:51–62.
